# Towards sustainable production and utilization of plant-biomass-based nanomaterials: a review and analysis of recent developments

**DOI:** 10.1186/s13068-021-01963-5

**Published:** 2021-05-06

**Authors:** J. Y. Zhu, Umesh P. Agarwal, Peter N. Ciesielski, Michael E. Himmel, Runan Gao, Yulin Deng, Maria Morits, Monika Österberg

**Affiliations:** 1 USDA Forest Products Laboratory, One Gifford Pinchot Dr, Madison, WI USA; 2 National Renewable Energy Laboratory, Golden, CO USA; 3 Renewable Bioproducts Institute, School of Chemical and Biomolecular Engineering, Georgia Institute of Technology, Atlanta, GA USA; 4 College of Materials Science and Engineering, Northeast Forestry University, Harbin, Heilongjiang China; 5 Department of Bioproducts and Biosystems, Aalto University, Espoo, Finland

**Keywords:** Lignin nanoparticles (LNPs), Cellulosic nanomaterials (CNMs), Cellulosic nano-whiskers (CNWs), Cell wall deconstruction, Fibrillation

## Abstract

Plant-biomass-based nanomaterials have attracted great interest recently for their potential to replace petroleum-sourced polymeric materials for sustained economic development. However, challenges associated with sustainable production of lignocellulosic nanoscale polymeric materials (NPMs) need to be addressed. Producing materials from lignocellulosic biomass is a value-added proposition compared with fuel-centric approach. This report focuses on recent progress made in understanding NPMs—specifically lignin nanoparticles (LNPs) and cellulosic nanomaterials (CNMs)—and their sustainable production. Special attention is focused on understanding key issues in nano-level deconstruction of cell walls and utilization of key properties of the resultant NPMs to allow flexibility in production to promote sustainability. Specifically, suitable processes for producing LNPs and their potential for scaled-up production, along with the resultant LNP properties and prospective applications, are discussed. In the case of CNMs, terminologies such as cellulose nanocrystals (CNCs) and cellulose nanofibrils (CNFs) used in the literature are examined. The term cellulose nano-whiskers (CNWs) is used here to describe a class of CNMs that has a morphology similar to CNCs but without specifying its crystallinity, because most applications of CNCs do not need its crystalline characteristic. Additionally, progress in enzymatic processing and drying of NPMs is also summarized. Finally, the report provides some perspective of future research that is likely to result in commercialization of plant-based NPMs.

## Background

Plant biomass is renewable and can be sustainably produced in large quantities in many regions of the world [1, 2]. Utilization of plant biomass to produce biofuels, biomaterials, and biochemicals to replace petroleum-based energy, materials, and chemicals is critically important for a future that employs a sustainable, effective circular economy. Plant biomass consists of three major components: cellulose (30% to 45% wt/wt), lignin (15% to 30% wt/wt), and hemicelluloses (15% to 35% wt/wt) [3, 4]. Internationally, considerable research effort has focused on the production of biofuels and biochemicals following the conversion of lignocellulosic plant biomass to fermentable sugars [5, 6] and aromatic compounds [7, 8]. This endeavor has been very challenging considering that plant biomass has evolved to resist biological deconstruction.

Wood is a major plant biomass that has been used traditionally as an economical source of material for large structures, such as buildings and bridges. Great commercial success has also been achieved by using wood to produce fibers, a polymeric material, for papermaking. With the exception of wood and bamboo, most plant biomass (i.e., herbaceous biomass and agriculture residues) do not have the strong structural integrity needed for construction applications. To achieve the goal of efficient utilization of herbaceous and agricultural plant biomass, we were compelled to learn from the successful papermaking industry. Rather than deconstructing lignocellulosic plant biomass to simple sugars and lignin aromatics, producing high-value nanoscale polymeric materials (NPMs), such as cellulose nanocrystals (CNCs), cellulosic nanofibrils (CNFs), and lignin nanoparticles (LNPs) also has potential to achieve commercial success. Here, we provide an overview of recent activities that support the production and applications of NPMs from lignocellulosic biomass. We also outline potential pathways to achieve sustainable production of NPMs based on existing understanding of the structure of the plant cell wall. The concept of sustainability is generally composed of three pillars—economic, environmental, and societal—which are required to meet present needs without compromising future needs. Here, we refer to the economic and energy-efficient production of plant-based NPMs using chemical and biological processes with low environmental impact.

**Fig. 1:**
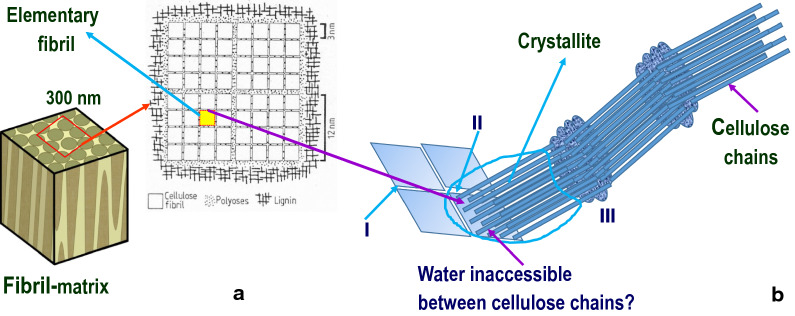
Schematics of cellulose ultrastructure: **a** radial aggregation in cell walls (through hydrogen bonding) of cellulose elementary fibrils into nanofibrils and fibril matrices [14] based on Fengel [11] (with permission from TAPPI ©); **b** longitudinal cellulose aggregation with crystalline and disordered regions based on Rowland and Roberts [17], I:coalesced surface with high order, II: readily accessible slightly disordered surfaces, III: readily accessible surfaces of strain-disorder tilt and twist regions

Plant biomass, such as wood, has a hierarchical structure in the radial direction. Specifically, each annual ring contains rows of wood cells corresponding to spring to fall growth. The cell wall contains the middle lamella (with high lignin concentration) and the primary and secondary cell walls (with highest concentrations of cellulose and hemicelluloses) [9]. NPMs are naturally embedded in the cell wall. Bundles of cellulosic fibrils in the secondary wall are composed of cellulose microfibrils, a term that has been commonly used in many textbooks and literature to refer to nanoscale fibrils of 10 to 20 nm in diameter [10–12], separated from lignin by xylan [13]. Moreover, the microfibril angle, the measure of microfibril orientation with respect to the cell longitudinal direction, dictates the cell or fiber stiffness. In the traditional terminology “microfibrils” became confusing when the recent concept of “cellulose nanofibrils” became popular. To be consistent with the physical dimension, we use the term of “microfibrils” in this review for microfibrillated cellulosic fibrils with dimensions from submicrons to a few micrometers. Nanofibrils consist of elementary fibrils and are crosslinked by hemicelluloses [14, 15]. Lignin is also present in the secondary wall displaying (1) primarily sub-nanometer physical contacts with hemicelluloses and (2) limited covalent bonding to hemicelluloses in the form of the lignin–carbohydrate complex (LCC). The majority of lignin and hemicelluloses tend to form a self-aggregated phase with limited interpenetration [13]. The presence of lignin further provides cell wall structural integrity and regulates the polarity and hydrophilicity of cell wall. Elementary cellulose fibrils consist of multiple (16 to 36) cellulose chains that appear to vary by plant species [11, 16–18] and are synthesized by plasma membrane-localized cellulose synthase complexes during plant tissue formation. Each cellulose chain is made of thousands of glucan units connected by the β(1–4) linkage. For wood, cellulose chain length, or degree of polymerization (DP), is on the order of 10,000 [19]. Cellulose chains are well organized and contain intra- and intermolecular hydrogen bonds (H-bonds) [19]; whereas the chain conformation is stabilized by the two types of intrachain H-bonds that provide cellulose with a stable structure, so that cellulose is difficult to dissolve in water and in many other solvents. Interchain hydrogen bonding aggregates elementary cellulose fibrils into larger fibrils. This structural diversity can be viewed at various length scales (or sizes) from a few nanometers (elementary fibrils), to tens of nanometers (nanofibrils), and finally to submicron fibrils (microfibrils) (Fig. 1**)**. Depending on the state of cellulose (e.g., natural state of cellulose in plants is cellulose I), the degree of hydrogen bonding and the local conformation of the C(6)H_2_OH group varies [15].

Understanding the native state supramolecular structure of cellulose is an on-going effort, even though cellulose was discovered nearly 180 years ago. For example, it is debatable whether or not water can penetrate into elementary fibrils or between cellulose chains, and how cellulose chains are aggregated in the longitudinal direction. Early models by Frey-Wyssling [10], Fengel [11], and Rowland and Roberts [17] nevertheless provided some reasonable pictorial understanding of these questions (Fig. 1). According to these models, the interior of elementary fibrils is inaccessible to water and only the surface of the elementary fibrils is accessible [10]. Furthermore, variations in cellulose accessibility to water along the cellulose chain direction indicate that certain regions in the fibrils are more organized, coalesced, or crystallized than others [10, 17] (Fig. 1b). The length of the crystallized regions is on the order of 30 nm in wood [11]. With this model, one can isolate an elemental crystallite from its natural state with a diameter of the elementary fibrils of 3 nm and a length of the ordered (crystal) cellulose chains of 30 nm using acid hydrolysis to cleave the disordered (i.e., defect or water-accessible) regions.

However, a recent study suggested that cellulose chains within elementary fibrils in untreated wood are indeed accessible to water [20]. This conclusion was based on several lines of evidence: (1) H_2_O-to-D_2_O exchange Raman studies indicated that signal intensity at 1380 cm^−1^ was more than what could be expected from various 18 to 36 chain crystal models. (The Raman band at 1380 cm^−1^ is due to CH_2_ bending mode of the C(6)H_2_OH group.) (2) The crystalline cellulose band at 93 cm^−1^ was absent. (3) The amount of *gt* conformation present was significantly higher compared to that of Avicel. (4) When conducting 64% sulfuric acid hydrolysis of loblolly pine wood, CNCs could not be produced. These results suggested that wood cellulose in its natural state was not crystalline. It was only upon hydrothermal treatment of wood that the cellulose became partly crystalline and CNCs could be produced [20]. Furthermore, crystallinities of CNCs produced from bleached pulp fibers using strong acid hydrolysis were not substantially higher than those of the original pulp fibers [21]. The small amount of increase in crystallinity, as measured by X-ray diffraction, is likely due to cellulose enrichment in the CNC samples after hydrolysis of amorphous hemicelluloses in the fibers. This raises an important question: can the crystallinity measurement method differentiate organized-but-not-crystalline from crystalline cellulose? It should be pointed out that crystallite length measured by Fengel [11] was from thermally treated wood. Agarwal and co-workers observed CNCs being produced only after the wood was first hydrothermally treated prior to acid hydrolysis under the same conditions applied to the untreated wood [20]. This finding suggests that structural consolidation plays a significant role in crystallizing natural cellulose [22]. This conclusion is also in agreement with small-angle neutron scattering studies that revealed cellulose chain consolidation, dehydration (hornification), and crystallization upon thermal treatment [23, 24]. It is also consistent with an early work by Battista that suggested cellulose crystallization by mild acid hydrolysis [25].

The above discussion indicates that the hierarchical structure of plant biomass requires some level of delignification followed by proper deconstruction of fibril structure to produce cellulose nanomaterials (CNMs). Depending on the process used for delignification, the side stream of dissolved lignin can be utilized to produce lignin nanoparticles (LNPs), another form of NPMs that has recently gained interest, in addition to CNMs, such as CNCs and CNFs. Although the sources of all these materials are renewable and low cost, achieving sustainable production of these NPMs is key to reaping the full benefits of using renewable natural plant biomass and achieving true sustainability.

## Lignin nanomaterials

### Molecular structure of lignin

Lignin is a polyphenolic polymer contained in vascular plants. In contrast to cellulose, lignin in native form is amorphous or not aggregated. Isolated lignin has a complex macromolecular structure that depends upon the source and isolation method [26]. Nevertheless, in general terms we note that the main building blocks of lignin, the monolignols, include *p*-coumaryl alcohol, coniferyl alcohol, and sinapyl alcohol that are connected by β-O-4, 5–5, β-5, 4-O-5, β-1, dibenzodioxocin, and β-β linkages [27]. Depending on the source of lignin, the composition of lignols and linkages between them vary.

### Lignin extraction methods

There are several industrial and laboratory-scale methods of lignin extraction and isolation from lignocellulosic biomass. Lignin is abundantly available as a side product from the pulping and biorefinery industry. However, because the objective of these processes is to liberate the cellulose and hemicellulose portion of the lignocellulosic biomass, these processes are harsh and lignin undergoes many chemical changes as a consequence [27].

Technical lignins extracted from the pulp and paper industry include kraft lignin (KL), lignosulfonates (sulfite pulping), and soda (or alkali, AL) lignin. KL and AL are produced by the alkaline pulping process, which uses aqueous sodium hydroxide and, in the case of the kraft process, also sodium sulfide. Lignosulfonate is produced by acidic pulping using excess aqueous bisulfite and sodium-, magnesium-, calcium-, or ammonium hydroxide. Following the pulping processes, lignins are dissolved in the pulping liquor and require extraction from the liquor for further use. These types of technical lignins are soluble in organic solvents or alkaline solutions. Lignosulfonates are soluble in water. Due to the prevalence of the kraft process, KL is the most abundant isolated lignin. Organosolv lignin (OSL) is obtained by the organosolv pulping process, which involves delignification at elevated temperatures using a mixture of water and organic solvents, such as ethanol or butanol, with a catalytic amount of acid [28]. OSL is of higher purity than KL, but it is currently available only at pilot scale.

Hydrolysis residual lignin of plant biomass from biorefineries is often insoluble in most solvents and may contain carbohydrate residues. The Bergius–Rheinau process employs concentrated hydrochloric acid for hydrolysis [29] to obtain lignin with high molecular weight. Dilute sulfuric acid hydrolysis of plant biomass or the Madison wood-sugar process [30] has now been replaced by enzymatic hydrolysis with a pretreatment or fractionation step. Enzymatic hydrolysis residual lignin represents a significant amount of biorefinery lignin, in addition to the lignin dissolved by the pretreatment or fractionation step, such as organosolv [31] and sulfite (SPORL) [32, 33]. Various fractionation or pretreatment process have been developed for biorefinery operations [34]. Combining hydrothermal treatment, commonly used for extraction of hemicelluloses [35], with extraction can recover lignin from fractionated solids [36, 37]; an example being aqueous acetone extraction [38].

Laboratory-scale production of lignin includes processes using ionic liquids [39], deep eutectic solvents [40], and molten salts [41]. These types of lignin are produced in small amounts and are generally not available to the broad research community. Hydrotropic fractionation using aromatic salts attracted great interest for wood pulping over a half century ago [42]. Recently, Zhu’s group at the USDA Forest Products Laboratory demonstrated rapid dissolution of plant biomass lignin at atmospheric pressure and ≤ 100 °C using recyclable acid hydrotropes, such as *p*-toluenesulfonic acid (*p*-TsOH) [43, 44] and maleic acid (MA) [45]. MA is an FDA-approved indirect food additive (21CFR175-177) with a low solubility at ambient temperature that eases recycle, therefore MA hydrotropic fractionation offers progress in biorefineries.

### Production methods for lignin nanoparticles (LNPs)

The chemical heterogeneity, broad molecular weight distribution, and low solubility of common types of commercially available lignin hinder its use in many applications. The preparation of LNPs with narrow size distribution and well-defined surface structure allows these problems to be overcome. This research field has gained increasing interest lately [46–49]. Additionally, due to their high surface area, this form of NPMs opens totally new application areas. Replacement of synthetic polymers by NPMs contributes to protection of the environment and thus further increases the value of LNPs.

Nanomaterials of different shapes can be prepared from lignin, including spherical LNPs, hollow LNPs, nanofibrils, nanosheets, and irregular LNPs [48]. Lignin nanomaterials possess most of the inherent properties of the original lignin, including antimicrobial, antioxidant, and UV shielding effects. Thus, these advantages are associated with the use of lignin nanomaterials. Spherical LNPs will be discussed in more detail in this and the following sections, whereas lignin nanofibers are addressed in Sect. 2.5. To the best of our knowledge, the first report of the preparation of lignin nanoparticles was published by Frangville et al. [50]. They prepared LNPs by dissolution in ethylene glycol followed by dialysis against water. However, these particles were irregular in shape. Particle shape, size, and surface topology play an important role in the application of nanoparticles, and uniform spherical particles have advantages in many applications. Qian et al*.* were the first to report the production of spherical LNPs using acetylated lignin to increase the solubility in tetrahydrofuran (THF) [51]. Inspired by this work, Lievonen et al. prepared an aqueous dispersion of spherical lignin particles from unmodified kraft lignin [52].

**Fig. 2:**
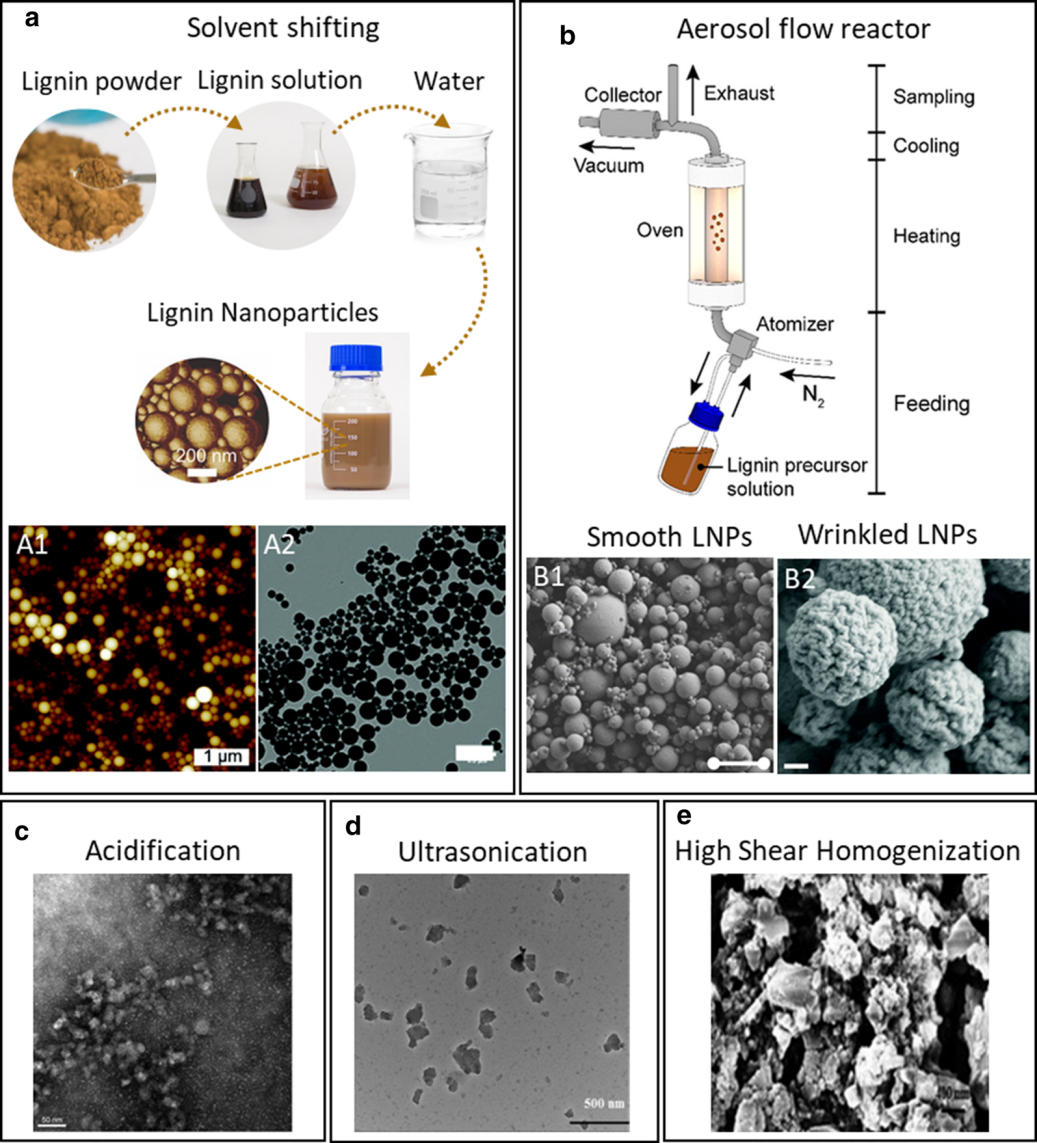
LNPs preparation methods. **a** Schematic of solvent shifting method; (A1) AFM height image of LNPs prepared by solvent shifting from Sipponen et al. [55]; (A2) TEM image of a CLP dispersion (scale bar 500 nm) from Lintinen et al. [63]. (A1) and (A2) reproduced by permission of The Royal Society of Chemistry. **b** Simplified experimental aerosol-flow reactor setup for the synthesis of lignin particles; (B1) SEM micrographs of solid lignin spheres synthesized by aerosol flow of OSL. **b **and (B1) Reprinted (adapted) with permission from Ago et al. [46]. Copyright © 2016, American Chemical Society. (B2) SEM image of wrinkled lignin particles with scale bars = 200 nm; From Kämäräinen et al. [70], reproduced by permission of The Royal Society of Chemistry. **c** TEM image of LNPs obtained by the acid precipitation of 0.56%wt Indulin AT in ethylene glycol and further dialysis in milli‐Q water with scale bars = 50 nm, reproduced with permission from Frangville et al. [50] © 2014 WILEY‐VCH Verlag GmbH & Co. KGaA, Weinheim. **d** Sonicated wheat straw lignin morphological characterization by TEM, reproduced with permission from Gilca et al. [73] Copyright © 2014 Elsevier B.V. All rights reserved. **e** SEM images of the lignin mechanically sheared for 2 h, reproduced with permission from Nair et al. [74] © 2014 Wiley‐VCH Verlag GmbH & Co. KGaA, Weinheim

There are many methods reported to prepare LNPs [49, 53] and while lignin source has recently been shown to affect the particle properties [54] the dominating factor is the chosen particle preparation method [49]. One commonly used method for preparation of LNPs is based on the dissolution of lignin in an organic solvent or water-organic solvent mixture followed by precipitation resulting from the increased concentration of water (antisolvent) (Fig. 2a) [52, 55–60]. In the literature, these methods are referred to as “solvent shifting”, “self-assembly”, “nanoprecipitation” or “solvent exchange” (Fig. 2a). Common to these approaches is that they result in stable aqueous dispersions of spherical, smooth LNPs. Final particle size and polydispersity depend upon the choice of lignin-dissolving solvent system. Dissolving lignin in a acetone:water mixture results in particles around 100 nm in diameter with narrow size range [61, 62]. In contrast, the use of THF [52] or THF:water:ethanol [63] solvents results in particles around 200 to 300 nm and slightly higher polydispersity. The three-solvent system (THF:ethanol:water) enables production of slightly more concentrated LNP dispersions than the two-solvent systems. Aqueous LNP dispersions are electrostatically stabilized due to charged, primarily carboxylic groups, present in lignin and decorating the particle surface. Hence, LNP dispersion stability is sensitive to pH and ionic strength. The colloidal stability of these particles and the size range of these particles has led some research groups to call them “colloidal lignin particles” instead of LNPs [63, 64]. The strategy of antisolvent addition also affects the aggregation tendency and final particle size. Rapid addition of water, or addition of lignin solution into water, has been found to lead to stable dispersion of small particles [64, 65]. In contrast, slow addition of water may lead to formation of larger aggregates. This effect of water addition rate was also observed in LNP production directly from wood using hydrotrope as solvent [66]. The interactions between lignin molecules and solvents are important for both dissolution and formation of nanoparticles. Recently, Wang and co-workers investigated the self-assembly and interactions of enzymatic hydrolysis lignin in organic–aqueous solvent mixtures using atomic force microscopy (AFM) and molecular dynamics simulations [67]. They showed that the hydrophobic skeleton of aromatic moieties interact with nonpolar solvents, whereas the hydrophilic carboxyl, and aromatic and aliphatic hydroxyl groups, interact with water. Consequently, a mixture of an organic solvent, such as THF or acetone, and water is most efficient for dissolving lignin. Furthermore, they showed that a shift towards pure water or pure organic solvents leads to self-assembly of spherical LNPs.

The reverse micelles formation method is based on the same principle as solvent shifting—that of self-assembly. In this method, particles are formed in a nonpolar solvent, permitting rearrangement of the hydrophilic groups to the “core” of the particle and formation of a “hydrophobic shell”. The obtained LNPs are spherical with a smooth hydrophobic surface. Zhou and co-workers reported preparation of LNPs in cyclohexane with average size of 130 nm and water contact angle of 89° [68]. This contrasts with the LNPs self-assembled in water that are generally hydrophilic.

Another popular method for LNP preparation is the acidification method based on a shift in pH [50, 69]. Lignin is dissolved at alkaline pH and precipitated by decreasing the pH. However, this mechanism of precipitation differs from solvent shifting. In contrast to solvent shifting, acidification results in protonation of carboxylic groups of lignin, which makes the acidic solution an antisolvent for the entire molecule and leads to precipitation of random aggregate-like structures. Because this is simple precipitation, contrary to the self-assembly of the solvent-shifting method, these particles do not form stable aqueous dispersions or well-defined spherical particles (Fig. 2c).

The solvent-shifting methods described above result in an aqueous dispersion of LNPs that can be dried after formation (e.g., by spray-drying) [63]. However, there are also methods directly resulting in dry particles. Ago and co-workers demonstrated that an aerosol-flow reactor can be applied to the preparation of dry LNPs from various types of soluble lignin [46]. In this method, spherical LNPs are formed in a two-step process. In the first step, lignin solution microdroplets are generated and then are dried in the second step. Strictly speaking, the process is a three-step process because lignin is dissolved prior to atomization. Nanoparticles produced by this method have a broad range of sizes (Fig. 2b). However, collection and separation of particles in a Berner-type low-pressure impactor allow quite narrow size fractions to be obtained. Kämäräinen and co-workers demonstrated the preparation of “wrinkled nanoparticles” (Fig. 2B2) using an aerosol-flow reactor [70]. They showed that the surface topology of dry LNPs produced by this method can be controlled by selection of solvent and use of a blowing agent. In this way, surface area can be controlled, which is of interest in many applications of LNPs. Mishra and co-workers suggested a slightly different method for preparation of LNPs using aerosol [71]. In their method, LNP droplets were frozen and then redispersed in water. This method is more complex than the methods described above but could be of interest for fabrication of hollow LNPs.

Acid hydrotropes have demonstrated robust performance in solubilizing lignin directly from plant biomass under atmospheric pressure and at low temperatures [43, 45], which provides opportunities to produce LNPs directly from plant biomass without using commercial technical lignin. LNPs can be produced by directly diluting the acid hydrotropic fractionation (AHF) liquor to below the minimal hydrotropic concentration [43, 72]. Resultant LNPs appeared to have an oblate spheroid shape with lateral size of nonaggregated particles of approximately 50 to 150 nm, but they have a tendency to appear in aggregates of lateral size around 300 to 400 nm [66]

Mechanical methods of LNP preparation include milling, ultrasonication, and high shear homogenization, applying mechanical disintegration of lignin macroparticles [73, 74]. This group of methods can be used for a broad spectrum of lignins. They also allow preparation of nanoparticles from insoluble lignin that is without chemical pretreatment. Nevertheless, the main drawback of these methods is the broad size distribution and the irregular and nonuniform structure of the resulting particles. The significant variability in surface topography and chemical structure is also problematic (Fig. 2d and e). Mechanical methods can furthermore cause chemical modification of lignin. Gilca and co-workers demonstrated depolymerization of lignin polymer chains and oxidative coupling of phenolic groups of lignin during preparation of LNPs by ultrasonication [73].

### Scalability of the LNP production processes

Some promising applications of LNPs include adhesives, biocomposites, and dispersion stabilizers. Table 1 lists the main LNP preparation methods, particle properties, and applications for the particles. These applications require production of large amounts of nanoparticles in a techno-economically feasible way. Nevertheless, most research to date has focused on small-scale production of particles, except for the work by Leskinen et al. [65] (who showed that 6 L of 2 wt% LNPs could be produced in one batch) and Lintinen et al. [63] (who demonstrated the production and further spray-drying of LNPs at similar scale, including recovery and reuse of solvents). Ashok and co-workers [75] and Abatti de Assis et al.[76] assessed the techno-economic feasibility of particle production based on solvent exchange and atomization, respectively; both processes were found to be scalable. The evaporation and circulation of solvents was found to be the most energy-consuming step [75]. Lourençon et al. recently showed energy savings in the atomization process by using acetone:water mixtures for lignin dissolution instead of previously used solvent systems [38]. Consequently, the choice of solvent system will be crucial for process feasibility. Another approach to scale-up of LNP production is the application of a continuous flow tubular reactor recently demonstrated by Ashok and co-workers [77]. The continuous flow tubular reactor represents a system of tubes with static mixing elements and a continuous flow of lignin solution into water during mixing. In this reactor, when lignin contacts water, a homogenous dispersion of spherical LNPs is spontaneously formed. Advantages of this approach include control over LNP size, continuous production of particles, scalability of the reactor, and energy efficiency [77]. Some mechanical methods, such as milling, can be used for large-scale production of LNPs as well. However, as mentioned earlier, drawbacks of these methods are the nonuniform particle shapes, broad size distributions, and heterogeneous surface chemistry and morphology of the resulting particles [48].

**Table 1:** LNP preparation methods

Method	Raw lignin	Solvent/antisolvent	Morphology, size	Surface properties	Applications	Refs.
Solvent shifting (nanoprecipitation, solvent exchange)	KL	Acetone and water/water	Spherical, *ca.* 100 nm	Hydrophilic, pH 4.3 ζ *ca.* -25 mV pH 3.9 ζ *ca.* -27 mV	Nanocomposites Pickering emulsions, drug delivery	[61, 62]
	KL		Spherical, *ca.* 244 nm	ζ *ca.* -37 mV	Component of biomaterial ink for 3D printing of scaffolds for cell culture	[78]
	KL		Spherical, *ca.* 109 nm, *ca.* 70.8 nm	ζ *ca.* -36 mV ζ *ca.* -37 mV	Water purification	[79]
	KL		Spherical, *ca.* 91 nm,	pH 4.0 ζ *ca.* -30 mV	Model surfaces	[80]
	KL		Spherical, *ca.* 97 nm,	ζ *ca.* -40 mV	Biocatalytic particles for SET-LRP, Pickering emulsions	[81]
	KL(+ BADGE)		Spherical, core–shell From 71 to 113 nm	ζ from *ca.* -32 mV to *ca*. -37 mV	Covalent surface modification, adhesives	[82]
	KL	THF and water/water	Spherical, 177–300 nm	Hydrophilic, smooth, ζ = 33–45 mV at pH 7	Pickering emulsions, immobilization of biocatalyst, adhesives	[55] [83] [84]
	KL		Spherical, *ca.* 142 nm	pH 3.9 ζ *ca*. -24.4	Model surfaces	[80]
	OSL		Spherical, smooth, aggregated, *ca*. 219 nm		Enzyme immobilization, biosensing	[85]
	KL		Spherical, 200–500 nm	Hydrophilic smooth, ζ *ca.* -60 mV		[52]
	Acetylated AL	THF/water	Spherical, 110 nm Spherical	Hydrophilic Hydrophilic	Potential in drug delivery and microencapsulation photo-protection agent	[51] [86]
	Organic acid lignin		100–600, 600–5000, 400–2000 nm			
	KL	THF and EtOH and water/water	Spherical, 200 nm	Hydrophilic smooth, ζ *ca.* -40 mV	Pickering emulsions, polymer composites	[63]
	Carboxylated KL	THF/water	Spherical, 167 nm		Biomedical applications, drug delivery	[87]
	EHL	Acetone and water/water or acetone	Spherical			[67]
	AL	EtOH and water/water	Spherical, 50–100 nm, 250–350 nm	Hydrophilic smooth, ζ ca. − 43 mV	Drug delivery	[64]
Reverse micelles	AL	Dioxane/cyclohexane	Spherical	Hydrophobic, smooth	Nanocomposites: UV-blocking, optimization of rheological properties	[68]
Acidification, pH shifting	KL (Indulin AT)	EG/HCl aq	Aggregate-like clusters	Uneven surface	Drug delivery, sorbents for heavy metal ions	[50]
		NaOH aq/HNO_3_ aq	Aggregate-like clusters	Uneven surface		
	KL	EG/ HNO_3_ aq	Aggregate-like clusters, 84 nm	uneven surface, ζ *ca.* -33 mV	Antimicrobial silver-infused nanoparticles	[69]
	KL	EG/HNO_3_ aq	Aggregate-like	uneven surface, partly hydrophilic,	Surface functionalization with, e.g., antimicrobial agents	[88]
	AL	NaOH aq/H_2_SO_4_ aq	Aggregate-like 768.4 ± 97.8 nm, 725.4 ± 51.3 nm	ζ *ca.* 2.8 mV ζ *ca*. − 13.4 mV	Emulsification, Pickering emulsion, template for synthesis of polymer capsules	[89]
Aerosol flow reactor, dry particles	Hydrothermal treatment	Acetone/none	Spherical	Smooth, hydrophilic, ζ *ca.* -35 mV		[38]
	KL, AL, OSL	DMF/none Water/none	Spherical, 30 nm-2000 nm	Smooth surface, hydrophilic KL ζ *ca.* -40 mV OSL ζ *ca.* -36 mV	Pickering emulsions	[46] [90]
	KL	DMF/none	Spherical, 50–2000 nm	Smooth	Coatings	[91]
Aerosol + freezing	AL	DMSO/water	Spherical particles and capsules, 80–200 nm	Smooth, hydrophilic, negatively charged	UV absorption, drug delivery	[71]
CO_2_ precipitation	KL	DMF/CO_2_	Coalesced quasi-spherical, *ca.* 38 nm	Uneven surface, hydrophilic	UV absorption	[92]
Mechanical treatment						
Sonication	AL	Water	Irregular, 10–50 nm	Uneven surface		[73]
Homogenization	KL	Water	Irregular, < 100 nm	Uneven surface	Nanocomposites: improvement of thermal and mechanical prop	[74]
Ball milling			Irregular, *ca.* 10 nm	Uneven surface		[48]
Low temperature milling			Irregular, ca. 10 nm	Uneven surface		[93]

### Production and applications of lignin nanofibers

While the spherical shape is a clear advantage of the LNPs in many applications, high aspect ratio is important in the applications of lignin nanofibers. Much of the work on lignin nanofibers has been focused on their use as precursors for carbon nanofibers [94–97]. Carbon fibers are very valuable for reinforcing of composites due to their high stiffness and strength combined with low density. Usually carbon fibers are made from synthetic and expensive polymers like polyacrylonitrile (PAN), but the high price of the polymer restricts the use of these fibers to mainly specialty applications. Due to its high carbon content, lignin has gained interest as a precursor for carbon (nano)fibers during the last few decades. Using lignin as precursor would not only reduce the dependence on fossil resources, but also reduce the price of the fibers by a factor of two [98]. However, it has been a challenge to achieve fibers with mechanical properties similar to fibers from PAN. In recent years carbon nanofibers have been prepared from various lignin sources, like organosolv lignin [99–101], kraft lignin [102–104] and lignosulfonate [105] and it has been shown that the chemical structure and molecular weight of the lignin has a strong influence on the final properties of the fibers, with high molecular weight and more linear structure leading to enhanced mechanical properties [106]. The preparation of carbon nanofibers is commonly achieved by electrospinning of melted lignin. To achieve good spinnability, the lignin is either chemically modified [101], fractionated [106] and/or mixed with binders [102, 107, 108]. Prior to carbonization at elevated temperatures, the process includes an oxidative stabilization step to prevent fusion of the fibers. Nevertheless, lignin-based carbon nanofibers have also been achieved without this step by addition of a small amount of CNCs [95]. Typical applications for the lignin carbon nanofibers include force reinforcement of composites and energy storage.

Due to their high surface area and the natural antioxidant properties of lignin, lignin fibers have also gained recent interest in biomedical applications. Wang et al. synthesized lignin–polycaprolactone (PCL) copolymers and mixed with PCL to produce a nanofibrous scaffold for cell culture [109]. The lignin–PCL copolymer enhanced the mechanical properties of the scaffold and interestingly, cell proliferation also increased. Similarly, Kai et. al., synthesized lignin poly(lactic acid) copolymers using acetylated lignin [110]. This copolymer was then blended with poly-l-lactide and nanofibers were produced using electrospinning. The lignin was able to hinder the oxidative stress induced by PLA and the produced scaffold demonstrated excellent antioxidant activity and biocompatibility.

Lignin nanofibers show great potential in biomedical applications, as well as for reinforcement of energy storage applications. However, in comparison to spherical LNPs, the production of high-quality nanofibers requires considerably more chemical modifications of the lignin or blending with synthetic polymers. For future applications, sustainability aspects of the process should be considered. The blending with cellulosic nanomaterials seems to be a promising route in this respect.

## Cellulosic nanomaterials (CNMs)

Currently, cellulose nanomaterials (CNMs) refers to mainly two types materials, i.e., CNCs produced primarily using concentrated sulfuric acid hydrolysis [21, 111, 112] by hydrolyzing disordered cellulose, and CNFs produced by mechanical fibrillation [113–115] to separate cellulose fibrils without or with a pretreatment step such as TEMPO-mediated oxidation [116], dilute acid [117] or enzymatic [118, 119] hydrolysis to decrease mechanical energy consumption for fibrillation. Using starting cellulosic materials containing lignin such as natural wood or unprocessed lignocelluloses [43, 120, 121] or unbleached chemical pulps [122–124], results in lignin-containing CNMs (LCNMs); e.g., lignin-containing CNCs (LCNCs) and lignin-containing CNFs (LCNFs). Using unprocessed lignocelluloses or natural wood has practical relevance to biorefinery operations. It should be pointed out that CNMs have DP over 100 which is two orders of magnitude greater than sugars, therefore, most deconstruction methods for pretreatment/fractionation used in sugar-based biorefinery, such as alkali [125], acid [117, 126], organic solvent [127, 128], oxidation [116, 129, 130], ionic liquid [131], deep eutectic solvents [132, 133], as well as enzymatic treatment including endoglucanase [118, 119, 134], xylanase [135, 136], and complex enzymes [137] have been successfully used for producing CNM. The key is to find a simple treatment with low cost and minimal environmental impact. Table 2 summarizes commonly used chemical and enzymatic treatment methods for producing a variety of (L)CNMs (subsequent mechanical fibrillation or sonication are required for producing (L)CNFs). It also provided qualitative assessment of chemical recovery and process impact on environment. For producing CNCs using the most commonly used conventional concentrated mineral acid hydrolysis, there have been several reviews [15, 138, 139]. This is also the case for producing carboxylated CNFs using TEMPO-mediated oxidation [140]. Here we will only focus on discussing some recent development using environmentally friendly chemicals and processes that are most promising to achieve sustainable production of CNMs that are relevant to biorefineries.

**Table 2:** A summary of common chemical and enzymatic treatment methods for producing (L)CNMs

Methods		Chemical	Chemical recovery, impact ^1^	Raw materials	CNM type	Surface groups	Refs.
Concentrated mineral acid hydrolysis		Sulfuric acid	−	Bleached wood pulp	CNCs	[HSO_3_]	[21, 112]
		Hydrochloric acid	−	Bleached softwood pulp	CNCs	None	[149]
		Phosphoric acid	−	Whatman paper	CNCs	[PO_4_]	[150]
		Sulfuric acid	−	Bleached wood pulp	CNCs + CNFs	[HSO_3_]	[126, 142]
		Sulfuric acid	−	Poplar wood	LCNCs	[HSO_3_]	[120]
Concentrated dicarboxylic acid hydrolysis		Oxalic acid or Maleic acid	+	Bleached wood pulp	CNCs + CNFs	[COOH]	[143, 148]
		Oxalic acid	+	Whatman paper	CNCs + CNFs	[COOH]	[151]
		Maleic acid	+	Unbleached hardwood pulp	LCNCs + LCNFs	[COOH]	[123, 124]
Acid hydrotrope		*p*-TsOH (aromatic sulfonic acid)	0	Undelignified birch fibers Wheat straw	LCNFs LCNFs	None None	[72] [44]
		Maleic acid	+	Poplar, birch wood Switchgrass	LCNFs LCNFs	[COOH] [COOH]	[45, 121] [152]
Dilute acid		Oxalic acid	0	Bleached wood pulp	CNFs	None	[117]
Oxidation		Ammonia persulfate	−	Variety cellulosic materials	CNCs	[COOH]	[130]
		Ammonia persulfate	−	Bleached birch pulp	CNFs	[COOH]	[153]
		Periodate + chlorite	−	Bleached wood pulp	CNFs	[COOH]	[129]
		TEMPO + NaBr + NaClO	0	Bleached wood pulp	CNFs	[COOH]	[140]
		TEMPO + NaBr + NaClO	−	Softwood mechanical pulp	LCNFs	[COOH]	[154]
Solvent	DES	Choline chloride + urea	0	Bleached birch pulp	CNFs	None	[132]
	GVL	GVL	0	Unbleached GVL pulp	LCNFs	None	[128]
	Organosolv	Ethanol + SO_2_	0	Wood	LCNCs, LCNFs	None	[127]
	Ionic Liquid	[BMIMCI]	0	Cellulose powder	CNFs	None	[155]
		[BMIM][HSO4]	0	Bleached wood pulp; microcrystalline cellulose	CNCs	None	[156]
		[EMIM][OAc]	0	Wood	LCNCs	None	[157]
Enzymes		Endoglucanase	~	Bleached wood pulp	CNFs	None	[118, 119, 134]
		Xylanase	~	Bleached wood pulp	CNFs	None	[135, 136]
		Complex enzymes	~	Bleached wood pulp	CNFs	None	[137]

^1^ − : difficult and negative impact; + : relatively easy and less impact; 0: moderate; ~ : benign and low dosage no need for recovery

### Integrated production of highly thermal stable and carboxylated CNFs with CNCs

Kinetic analysis [141] and mineral acid hydrolysis experiments [142] indicate that using concentrated mineral acid hydrolysis under mild conditions for producing CNCs can result in substantial amounts of cellulosic solid residues (CSR) rather than soluble sugars. This substantially reduced cellulose loss to sugars that is difficult to recover and improved cellulosic solids yield. The CSR are partially hydrolyzed and depolymerized cellulosic fibers that can be easily fibrillated into CNFs with low energy input [126, 142]. This observation presents the opportunity to produce both CNFs and CNCs in one production line [142]. The amounts of CNFs and CNCs (or ratio of CNFs to CNCs) can be tuned by adjusting the acid hydrolysis severity, specifically acid concentration, temperature, and/or reaction time. Furthermore, the morphology of the CNFs can also be tailored by tuning the acid hydrolysis severity in addition to the extent of mechanical fibrillation [126, 142].

**Fig. 3:**
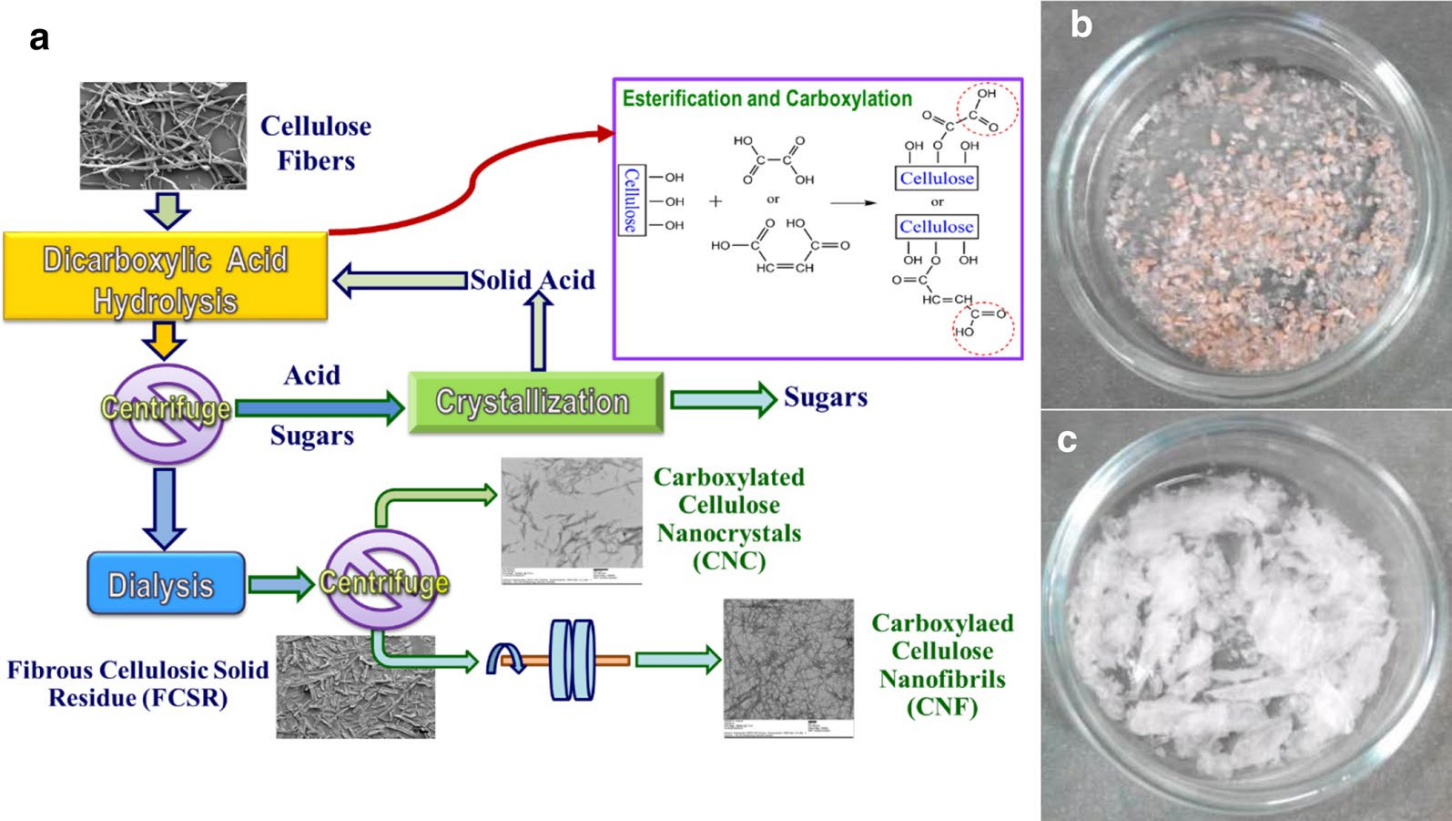
Concentrated solid dicarboxylic acid (DCA) hydrolysis for integrated production of highly thermal stable and carboxylated DC-CNCs and DC-CNFs with acid recovery. **a** Schematic flow diagram; **b**, **c** A comparison of thermal stability of DC-CNCs after heating at 105 °C for 4 h with S-CNCs from S64T45t45 (**b**: 64 wt% sulfuric acid at 45 °C for 45 min) with that from O70T100t60 (**c**: 70 wt% oxalic acid at 110 °C for 60 min). Reproduced with permission from Chen et al. [143] © The Royal Society of Chemistry

Using acids with low solubility in water or solid acids at ambient condition can substantially facilitate acid recovery. Based on the concept of “mild” acid hydrolysis to achieve integrated production of CNCs and CNFs in one production line as discussed above, weak acids can be used to reduce acid hydrolysis severity. Dicarboxylic acids (DCAs), i.e., OA (oxalic acid), MA (maleic acid), ScA (succinic acid) are solid acids with lower acidity than sulfuric acid. OA and MA have been evaluated for integrated production of CNFs with CNCs as shown in Fig. 3a [143, 144] to ease acid recovery. To compensate for the loss of reaction severity using weak dicarboxylic acids, the reaction temperature was raised to approximately 100 to 110 °C. With acid concentration at 50 wt% or higher, the acid solution will not boil at 110 °C due to the high acid concentration. Therefore, acid hydrolysis can be carried out at atmospheric pressure using inexpensive reactors to substantially decrease capital cost compared with conventional concentrated sulfuric acid hydrolysis.

The resultant DC-CNCs were highly thermal stable, as shown by comparing S-CNCs (concentrated sulfuric acid hydrolysis CNCs) to those from O-CNCs (oxalic acid hydrolysis CNCs) under heating (Fig. 3b and c). This is partially due to higher crystallinity and greater DP [145] of the resultant O-CNCs than S-CNCs. Typically, the weaker acidity of DCAs tends to result in longer DC-CNCs of about 500 nm and a lower DC-CNC yield of approximately 20% [143]. Most of the remaining material is partially hydrolyzed CSR. The CSR can be mechanically fibrillated into CNFs to achieve integrated production of DC-CNCs with DC-CNFs [143]. Reaction severity-based kinetics was developed to tune the yields and morphologies of DC-CNCs and DC-CNFs [146]. When using concentrated MA hydrolysis of bleached kraft eucalyptus pulp (BEP) fibers, cellulose DP of the hydrolyzed fibers can be expressed using a modified combined hydrolysis factor for glucan, *CHF*_G_, as a measure of reaction severity:

1 $${CHF}_{G}=\text{exp}\left(\alpha "-\frac{E"}{RT}+\beta "{C}^{\varepsilon }\right)C\cdot t$$

2 $$\frac{DP}{{DP}_{BEP}}={F}_{DP}{e}^{-j\bullet {CHF}_{G}}+{S}_{DP}{e}^{-{CHF}_{G}}+\left(1-{F}_{DP}-{S}_{DP}\right),$$

where α”, β”, and *ε* (exponential index) are adjustable parameters, *E*” is apparent activation energy (J/mol), *R* = 8.314 (J/mol/K) is universal gas constant, *T* is reaction temperature in Kelvin, *C* is acid concentration in mol/L, and *t* is reaction time in min. ${DP}_{BEP}$ is the DP of feed BEP fibers, ${F}_{DP}$ and ${S}_{DP}$ are the respective fraction of cellulose depolymerization contribution from fast and slow reaction cellulose, *j* is ratio of the reaction rates between the rapid and slow depolymerizing cellulose fractions. The concept of level-off cellulose DP (LODP) is well known [147]. Here the LODP is represented by the balance of depolymerization contributed by the fast and slow cellulose, i.e.,$LODP/{DP}_{BEP}=(1-{F}_{DP}-{S}_{DP})$. The measured DP data of hydrolyzed BEP samples were fitted to Eqs. (1) and (2) to obtain α” = 39.43, β” = 0.373 (L/mol)^ε^, ε = 0.5, E = 143,000 (J/mol), ${F}_{DP}$ = 0.467, ${S}_{DP}=0.328$, and *j* = *58,* for MA hydrolysis of BEP fibers [146]. LODP was obviously reached using concentrated MA hydrolysis.

**Fig. 4:**
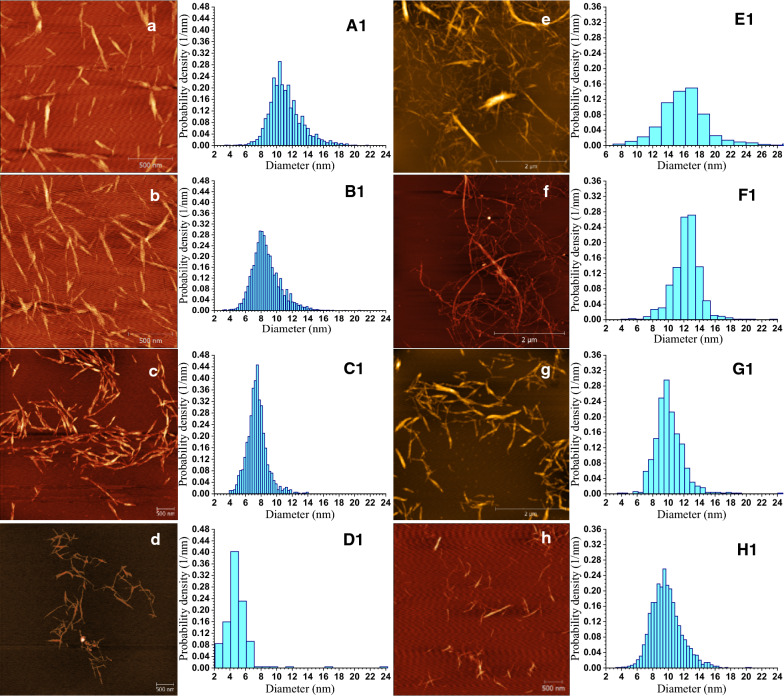
AFM images and AFM-measured height distributions of concentrated maleic acid (MA) hydrolysis M-CNCs (**a**–**d**) and M-CNFs (**e**–**h**) produced from bleached eucalyptus pulp fibers under various concentrated MA hydrolysis severities: *CHF*_G_ = 1.0, 4.1; 6.8, 20.4 for **a**–**d** (CNCs), respectively; *CHF*_G_ = 0.01, 0.09; 1.12, 2.04 for **e**–**h** (M-CNFs), respectively. The M-CNF shown in **e**–**h** were produced using only one pass through a microfluidizer. Scale bar = 500 nm for **a**–**d** and **h**, = 2 μm for **e**–**g**. Reproduced with permission from Wang et al. [146] © Wiley‐VCH Verlag GmbH & Co. KGaA, Weinheim

The morphologies of MA CNCs (M-CNCs) and M-CNFs were found to correlate well with hydrolysis severity *CHF*_G_, i.e., a higher severity results in shorter and thinner M-CNCs and less entangled M-CNFs or even individually separated M-CNFs [146, 148]. This observation is clearly supported by Fig. 4. Because DP of the acid hydrolyzed fibers can be accurately predicted from Eq. (2) using *CHF*_G_. It can be restated that for concentrated dicarboxylic acid hydrolysis, DP can be used as a control parameter for the integrated production of DC-CNCs and DC-CNFs.

In addition to the potential of achieving sustainability, tailoring CNM morphology, and surface carboxylation, using recyclable dicarboxylic acids for integrated production of CNCs with CNFs (or lignin-containing CNCs with lignin-containing CNFs, to be discussed later) also has the advantages of (1) reducing capital investment and operating cost by simultaneously producing CNCs and CNFs in one production line without establishing two very different production facilities, (2) tuning the product ratio of CNCs over CNFs by adjusting the hydrolysis severity to meet market demands for CNCs and CNFs (The low CNC yield from dicarboxylic acids is advantageous because CNC market is substantially smaller than the demand for CNFs), and (3) achieving rapid production of cellulosic nano-whiskers (CNWs) with morphology similar to CNCs (a materials with growing market to be discussed in the following subsection) by simply eliminating the CNC separation step using dialysis to feed all hydrolyzed fibers into mechanical fibrillation.

### Cellulosic nano-whiskers (CNWs) with CNC-like morphology

At high hydrolysis severities using MA [146] or using strong acid, such as sulfuric acid [126, 142], the resultant CNFs from mechanically fibrillating the hydrolyzed CSR can be individually separated nano-whiskers (Fig. 4h) with a morphology similar to CNCs (Fig. 4a–d). The nano-whisker sample shown in Fig. 4h actually has a lower crystallinity than the corresponding CNCs because it was obtained after mechanical fibrillation [126, 148]. Therefore, this type of individually separated nano-whiskers with morphology similar to CNCs may not be called CNCs. Here we use the term cellulose nano-whiskers (CNWs), the term used in some literature [158] to describe this class of CNMs with morphology and surface charge properties similar to those of CNCs, but without consideration of crystallinity, i.e., CNWs may or may not have the crystalline property that CNCs have. This enables production flexibility by using less harsh conditions and easily recyclable chemicals or low-cost enzymes to achieve sustainability. For example, because of low CNC yield when using dicarboxylic acids especially at low fractionation severities, the dialysis step shown in Fig. 3a can be eliminated and simply feed all hydrolyzed cellulosic solids (CNCs + CSR) into mechanical fibrillation to produce CNWs. The wide availability of S-CNCs in the market place, thanks to several pilot facilities and one commercial facility, resulted in the phenomenon that S-CNCs have been unnecessarily used for many applications that do not need crystalline cellulose, such as rheology modifiers [159–161], hydrogels [162–164], and 3D printing [165–170], where CNWs are adequate. This inappropriate use history has commercial implications because S-CNCs are an expensive material due to the difficulties in economic recovery and the corrosive nature of sulfuric acid at very high concentrations. Therefore, making use of CNWs to distinguish morphological dimensions from crystallinity is very important. CNCs can be considered as a subcategory of CNWs. To most people in the CNM community, CNWs and CNCs are used interchangeably. However, the rational to differentiate these two terms is to provide flexibility to achieve production sustainability to facilitate commercialization.

**Fig. 5:**
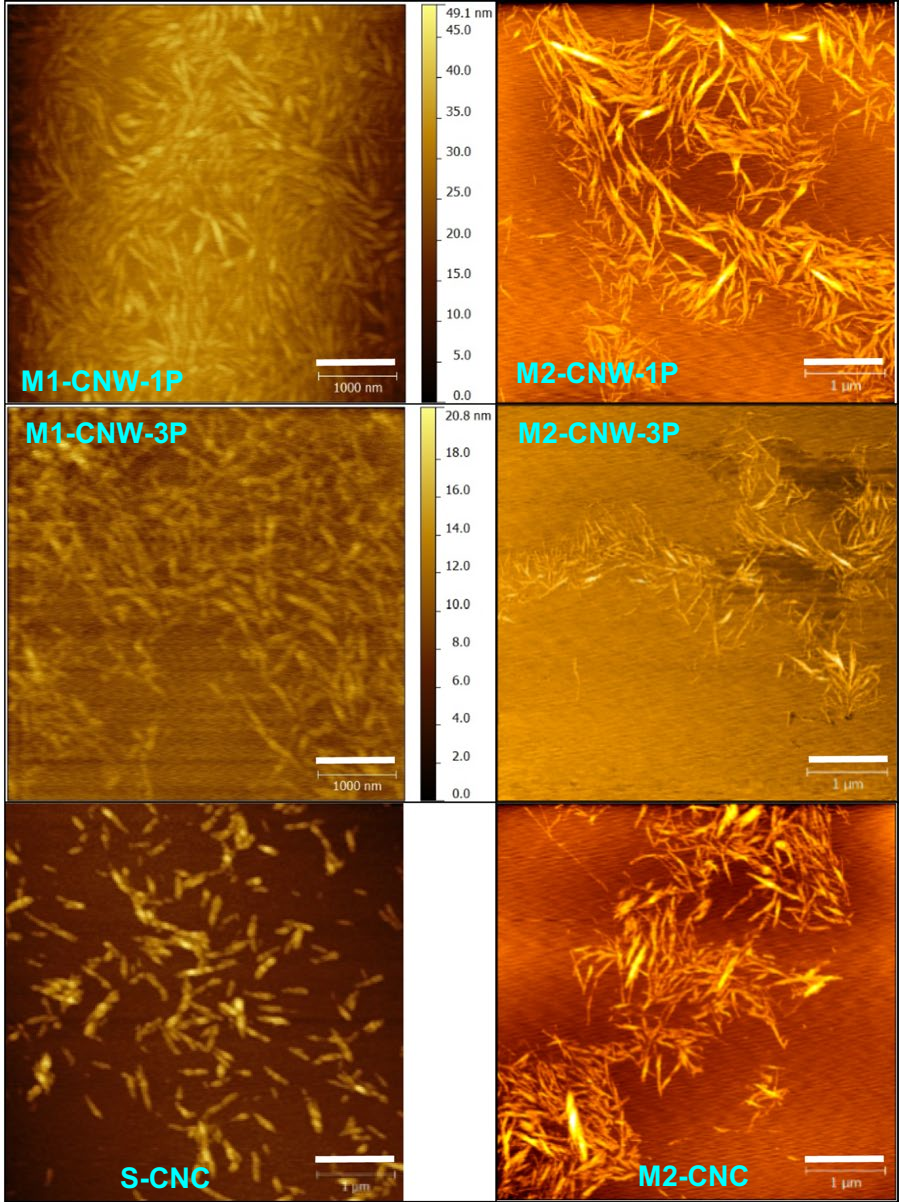
AFM images of cellulosic nano-whiskers (CNWs) with CNC-like morphology produced by concentrated MA hydrolysis of bleached eucalyptus pulp fibers (BEP) followed by mechanical fibrillation of the hydrolyzed cellulosic solids residue (CSR), in comparison with CNCs. All scale bars = 1000 nm. Top and middle rows: morphologies of CNWs from two high severities pilot-scale runs (left column M1: M70T120t120, right column M2: M75T100t120). Top row: 1 pass homogenization; middle row: three passes homogenization. Bottom row: morphologies of CNC from concentrated acid hydrolysis (left: sulfuric acid hydrolysis of spruce dissolving pulp from FPL pilot plant, right: MA hydrolysis of BEP under M2). Reproduced from Wang et al. [148]

To ensure the production of CNWs after mechanical fibrillation, DP of DCA-hydrolyzed CSR should be controlled to 250 by using a proper hydrolysis severity *CHF*_*G*_ (Eqs. (1–2)). Scale-up of this production process, from 5 g [146] (Fig. 4) to 750 g [148] (Fig. 5), using *CHF*_*G*_ as a scaling factor showed excellent scalability. It is interesting to note from Fig. 5 that one pass through homogenization was sufficient, suggesting that the energy cost for mechanical fibrillation is low. Comparing run M1 with M2 in Fig. 5 and the results in Fig. 4 (right panel) suggests that a minimal *CHF*_*G*_ ~ 3 is required to produce CNWs from bleached kraft eucalyptus pulp fibers. Figure 5 clearly shows that the morphologies of the CNW samples are very similar to M2-CNC (CNCs were separated in run M2 according to Fig. 3a) from the same feed fibers and using the same MA in hydrolysis. This type of CNWs, specifically M2-CNW-3P, has been successfully demonstrated as a substitute for S-CNCs in reinforced unsaturated polyester composites with equivalent or better performance and higher thermal stability [171] (note that M2-CNW-3P was incorrectly labeled as M-CNC in the publication [171]), as well as a rheology modifier in water-based drilling fluid study for improved filtration efficiency and thermal stability [172].

### Producing lignin-containing cellulose nanomaterials

Lignin containing cellulose nanofibrils (LCNFs) have been produced simply by mechanically fibrillating unbleached pulp fibers [122, 173] or with chemical treatment to depolymerize cellulose to facilitate fibrillation [123]. However, from the view-point of biorefinery, raw lignocellulosic materials should be the feedstock for producing lignin-containing cellulose nanomaterials (LCNMs), rather than commercial pulp fibers. Lignin brings several unique properties to LCNMs, such as hydrophobicity and UV light protection. LCNMs have attracted great interest recently. Partial delignification is necessary to facilitate LCNF production from raw lignocelluloses [72, 173]. Many fractionation processes, such as conventional alkaline or sulfite pulping, organosolv solvents [127, 128], ionic liquids [39], deep eutectic solvents [40], are all capable of delignification. Even concentrated sulfuric acid hydrolysis was capable of producing LCNCs with very high lignin content of approximately 30% from Wiley-milled wood after hydrothermal treatment [120]. The key is to use the most sustainable process to achieve commercial success. Among many fractionation processes, acid hydrotropic fractionation (AHF) demonstrated by Zhu and co-workers at the USDA Forest Products Laboratory using *p*-TsOH [43] and especially MA [45] has several advantages: (1) substantial and rapid delignification at atmospheric pressure and below the boiling point of water to reduce capital and operating costs; (2) ease in chemical recovery as these two acids are solid acids with low solubility in water at ambient condition; (3) MA is an FDA-approved indirect food additive (21CFR175-177) (Code of Federal Regulations (CFR)) with minimal environmental impact; (4) lignin separation can be achieved simply by diluting the fractionation liquor with water to below the minimal hydrotropic concentration (25 wt% for MA) and the dissolved lignin has low degree of condensation to facilitate valorization; (5) the dissolved hemicellulosic sugars can be directly dehydrated into furan using the acid remained in the fractionation liquor without additional catalysts. All these advantages fit well to sustainable biorefinery operation to valorize all major components of lignocelluloses.

Producing LCNFs directly from poplar [45] and birch wood [72, 121], wheat straw [44], switchgrass [152] using AHF have been demonstrated. The degree of delignification can be controlled by a combined delignification factor, *CDF* [44, 174], as shown in Eqs. (3) and (4), whereas the amount of hemicellulose dissolution can be controlled by a combined hydrolysis factor for xylan, *CHF*x [44, 146], as shown in Eqs. (5) and (6).

3 $${{CDF}}=\text{exp }(\alpha ^{\prime}-\frac{{E}^{^{\prime}}}{RT}+\beta ^{\prime}C)\cdot C\cdot t$$

4 $${L}_{\text{R}}=\left(1-\theta ^{\prime}-{\theta ^{\prime}}_{\text{R}}\right){e}^{-{{CDF}}}+\theta ^{\prime}\cdot {e}^{-f^{\prime}\cdot {{CDF}}}+{\theta ^{\prime}}_{\text{R}}$$

5 $${{CHF}}_{X}=\text{exp}(\alpha -\frac{E}{RT}+\beta C)\cdot C\cdot t$$

6 $${X}_{R}=\left({1}-\theta-{\theta }_{\text{R}}\right)\text{exp}\left(-{{CHF}}_{X}\right)+\theta \text{exp}\left( -f \cdot {{CHF}}_{X}\right)+{\theta }_{\text{R}}.$$

Again, α, α′, β, β′ are adjustable parameters similar to α” and β” in Eq. (1). *E* and *E*′ are apparent activation energy (J/mol). *R* is universal gas constant. *C* is acid concentration in mol/L. T and *t* are reaction temperature and time in Kelvin and min, respectively. θ and θ′ are the initial fractions of slow reacting xylan and lignin, respectively. *f* and *f* ′ are the ratios of the reaction rates between the slow and fast xylan and slow and fast lignin, respectively. θ_R_ and θ_R′_ are the residual xylan and lignin, respectively. All the adjustable parameters along with activation energy *E* and *E*′, θ, θ′, *f*, *f* ′, θ_R_, θ_R′_ are obtained by fitting the experimentally measured xylan and lignin dissolution data to Eqs. (3–6) as demonstrated [44, 148, 152].

**Fig. 6:**
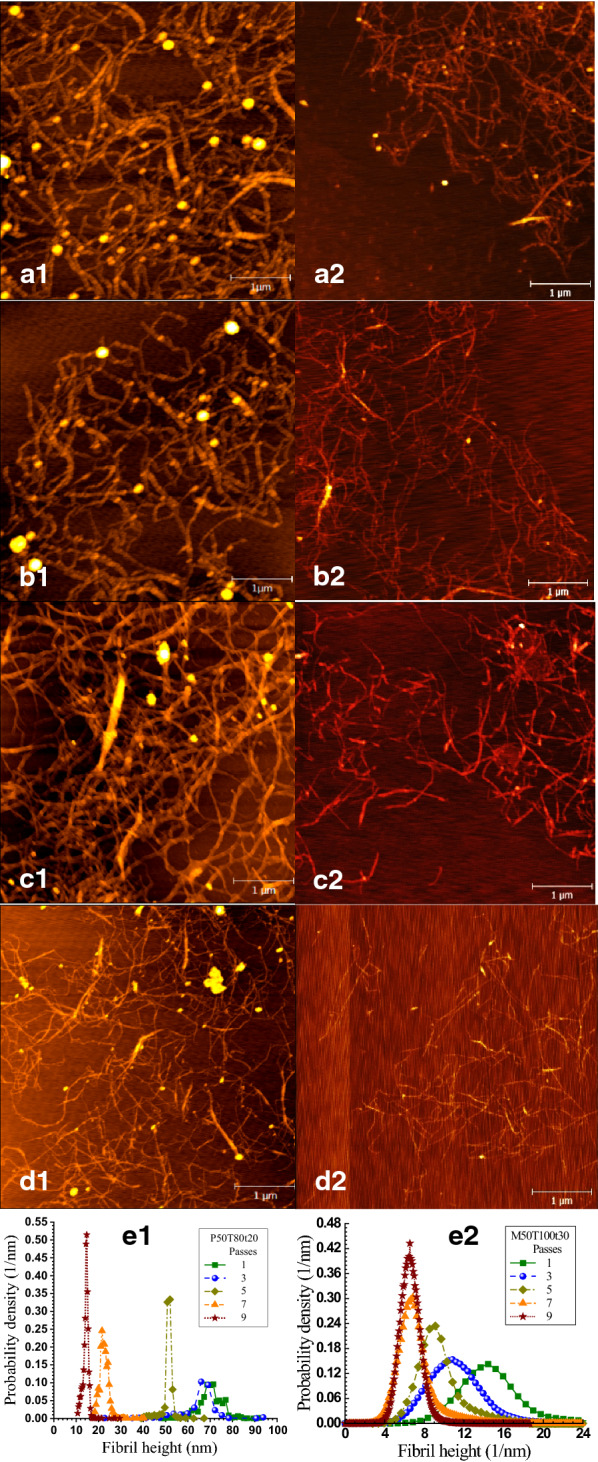
AFM images (**a1**–**d2**) and AFM-measured height distributions (**e1** and **e2**) of CNFs from concentrated *p*-toluenesulfonic acid (left panel, from Bian and co-workers [72]) and maleic acid (right panel, from Cai and co-workers [45]) hydrotropic fractionated birch wood solids. Reproduced with permission from The Royal Society of Chemistry©

Very uniform LCNFs were produced (Fig. 6) from fractionated birch wood solids of lignin content approximately 16%, even with minimal mechanical fibrillation (one pass through microfluidization) and therefore minimal energy for fibrillation. By comparing birch wood LCNFs (Fig. 6) from *p*-TsOH fractionation [72] with those from MA fractionation [45, 121], the advantages of MA fractionation is obvious, i.e., much thinner LCNFs suggesting easier to fibrillate [45, 121]. This is due to lignin and cellulose carboxylation which, respectively, enhanced the lignin lubrication effect and reduced hydrogen bonding among cellulose fibrils [45, 121, 175]. Carboxylation also provides the resultant LCNFs with higher degree of charge for dispersion.

The potential of AHF fractionation using MA for biorefinery operation was also demonstrated in a recent study [121]. In addition to minimal energy input for producing carboxylated LCNFs as presented above, the carboxylated solids from MA AHF are readily digestible even at a low cellulase dosage of 10 FPU/g glucan due to substantial decrease in nonproductive cellulase binding to substrate lignin, achieved through pH-mediated electric repulsion between cellulase and carboxylated (charged) substrate at elevated pH of 5.5–6.0 [176, 177]. The dissolved lignin has low degree of condensation which facilitated catalytic conversion to monophenols with good yield [121]. Furthermore, the dissolved xylose was converted to furfural using the MA in the fractionated liquor at good yield of 70%. The MA is than recovered as discussed in the following subsection.

### Acid recovery

The recyclability of the solid acids especially MA discussed above, was also demonstrated. An early study simply reused the *p*-TsOH fractionation liquor [43]. The study found that the chemical compositions of the fractionated solids from using fresh liquor are similar to those from the recycled liquors under different fractionation conditions. In another study using MA [45], the dissolved lignin in the fractionation liquor was first precipitated after diluting the MA concentration to 15 wt% (below the minimal acid hydrotropic concentration of 25 wt%). The lignin precipitated liquor was dehydrated at 180 °C for 10 min, which resulted in a furfural yield of 70% based on the amount of xylan dissolved in the liquor. The furfural distilled liquor with MA concentration of 50% was reused multiple times for fractionation after spiking 5% of the initial amount of MA (assuming 5% loss that include the amount of MA remained in the fractionated solids). The chemical composition of the fractionated solids from using fresh MA liquor were essentially identical to those from using recycled liquors.

**Fig. 7:**
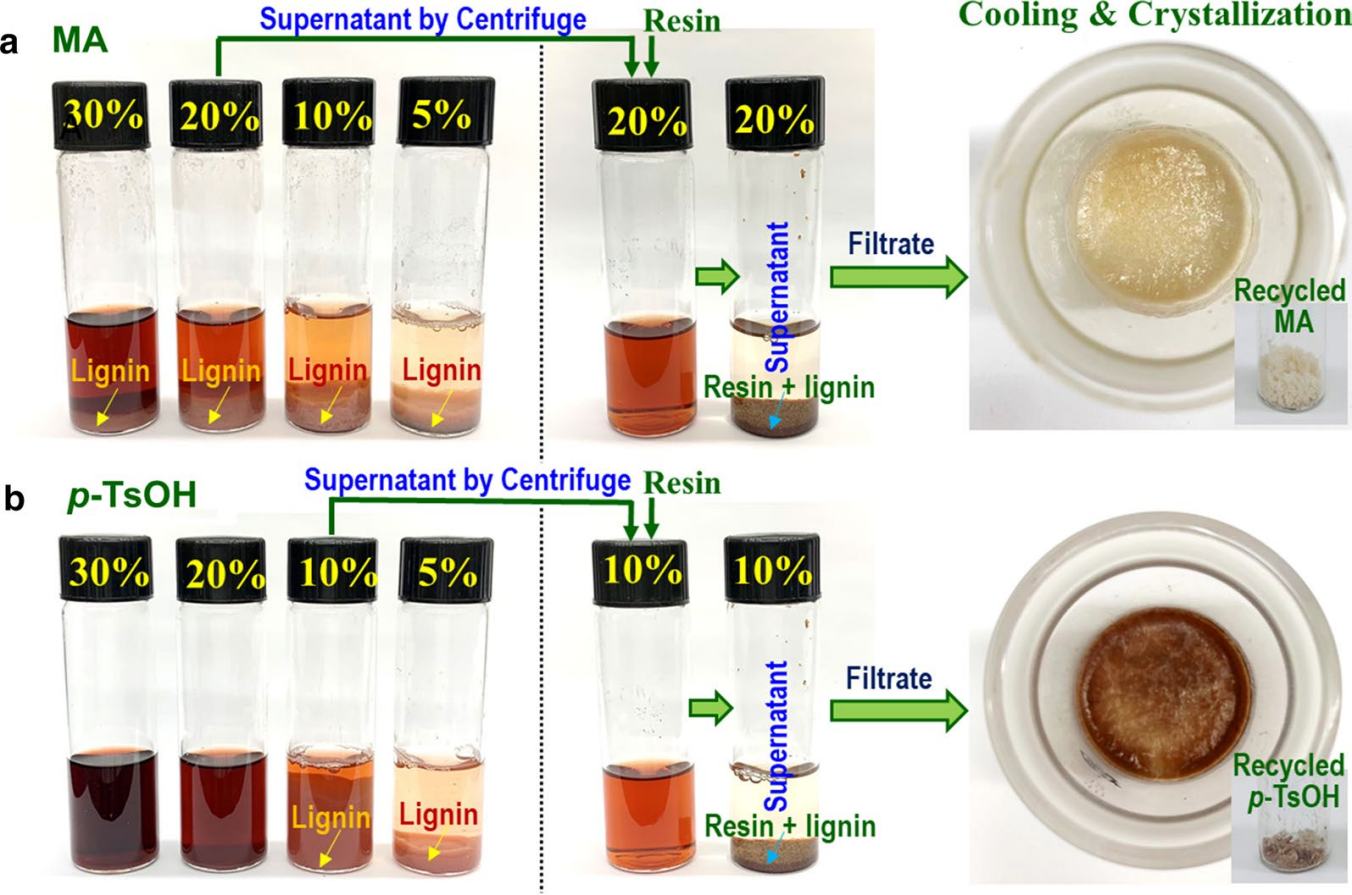
Fractionation liquor dilution to precipitate lignin, followed by resin adsorption of residual dissolved lignin for recovering acid by crystallization through cooling after evaporation to re-concentrate. Reproduced with permission from Cai et al. [121] Wiley–VCH GmbH & Co. KGaA, Weinheim

A separate laboratory study the recoveries of MA and *p*-TsOH were quantified and compared [121]. As shown in Fig. 7, the MA and *p*-TsOH fractionation solids were separated from the liquors through filtration with minor washing to achieve a diluted liquor of 30% acid concentration. The MA and *p*-TsOH liquor were then diluted to 20% and 10%, respectively, to achieve approximately 80% lignin precipitation. After extracting the remaining lignin using resin, the diluted acid solution was crystallized at 60 °C at atmospheric pressure. The acid recovery of 87% and 76% for MA and *p*-TsOH were determined gravimetrically with purities of approximately 95% for both acids. This recovery did not account for any acid remained on the fractionated solids due to incomplete washing. This suggests that the recovery of these two solid acids is much easier than soluble sulfuric acid. This is especially true for MA that has low acidity and therefore, less corrosive to materials, and low solubility at the ambient condition.

## Enzymatic processing—emerging research associated with biorefinery

### Cellulose depolymerization: modes of action

**Fig. 8:**
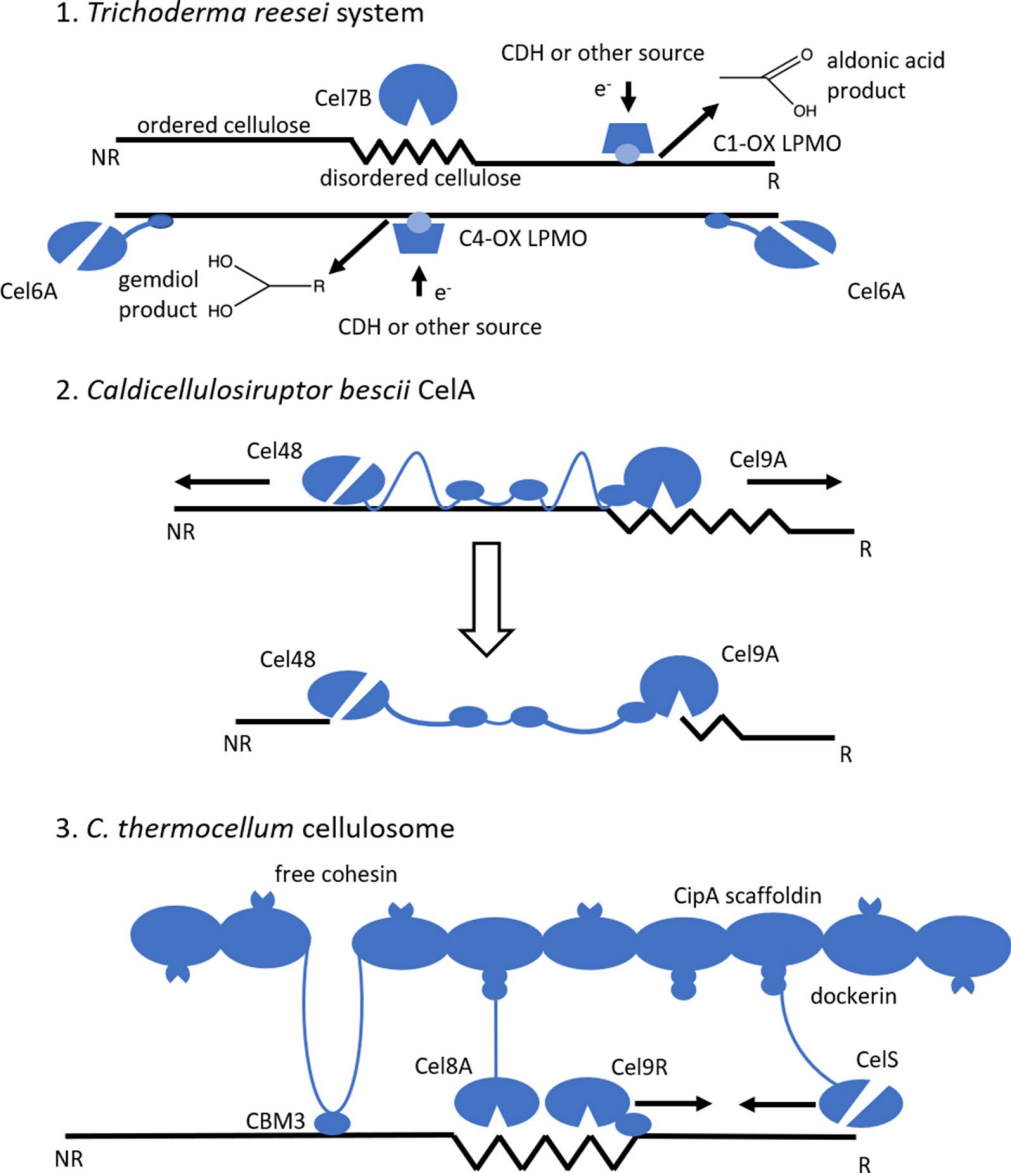
Shown are model representations of the (1) Mono-functional system found in cellulolytic fungi (i.e., Trichoderma reesei): Cel6A and Cel7A are processive cellobiohydrolase that initiate from the non-reducing and reducing ends of cellulose chains, respectively. Cel7B is an endoglucanase which hydrolyzes cellulose at mid-chain positions thus producing new chain ends for cellobiohydrolases to initiate hydrolysis. CDH is a cellobiose dehydrogenase which acts as a redox partner in the LPMO mechanism. (2) The multi-functional system used by some cellulolytic bacteria (i.e., *Caldicellulosiruptor bescii*) consists of a GH9 endoglucanase catalytic domain and a GH48 exoglucanase domain and also contains three cellulose-binding domains. This bacterial cellulase is one of the most effective enzyme systems ever reported for degrading cellulose and exhibits a “pit-digging” mechanism as shown in reference [203]. (3) The highly aggregated cellulosome consisting of various cellulase and cellulose-binding domains bound to a protein scaffold by the dockerin–cohesin interaction. This diagram represents the canonical *Clostridium thermocellum* CipA scaffolding structure containing nine type I cohesin domains (type II cohesin domains are not shown). Depicted are a CBM3 cellulose-binding domain, a Cel8A endoglucanase, and a CelS exoglucanase. This system works synergistically with free enzymes such as Cel9I, a processive endoglucanase. A detailed description of these enzyme systems is presented in reference [178–180]

Cellulases typically found in the mono-functional enzyme system are defined as the cellobiohydrolases (CBHs), endoglucanases (EGs), and β-d-glucosidases. CBHs are processive enzymes that hydrolyze cellulose from specific cellulose chain ends, whereas EGs hydrolyze cellulose chains randomly (Fig. 8). Cellobiases hydrolyze cellobiose to glucose, which prevents CBH end-product inhibition. The second class of cellulases includes the multi-functional enzymes, which are single gene products composed of two or more catalytic activities. The highly aggregated enzymes (cellulosomes) constitute the third major class of cellulose-degrading enzymes. These enzymes are usually of high molecular weight and have one or several CBMs [178–180].

Until relatively recently, enzymatic schemes different from the well-studied hydrolytic mechanisms were not reported. In 2010, Vaaje-Kolstad and co-workers made it clear that the microbial world harbored another tool kit for deconstructing cell wall and even solubilizing polymers; this mechanism was oxidative and not hydrolytic [181]. The report of lytic polysaccharide monooxygenases (LPMOs) has now significantly revised our view of plant biomass biodegradation. LPMOs oxidatively fragment the glycosidic bonds over an ever-increasing range of polysaccharides, including crystalline cellulose, disordered cellulose, carboxymethylcellulose, mixed β-(1,3;1–4) glucans, xyloglucans, glucomannan, xylan, and xylo-/mannodextrins [182]; however, this list of target substrates for LPMOs is likely to grow. It is also important to note that some LPMOs have preference of crystalline versus disordered cellulose and may prefer certain cellulose allomorphs [183, 184]. Such characteristics are very useful for planning deconstruction schemes for CNC or CNF production. LPMOs are regioselective regarding their mode of action on the C1 and C4 carbon bonds. Oxidation by LPMOs of β-(1,4)-linked glucans at either the C1 or C4 carbon positions generates mixed non-oxidized and C1 and C4-oxidized products. Moreover, C4-oxidation forms 4-ketoaldoses (gemdiols) from the original ketone carbonyls. C1-oxidation forms labile δ-lactones that dissociate in water to form aldonic (carboxylic) acids that are not fermentable by yeast or bacteria [185, 186].

### Novel cellulase development

The objective of modern biorefineries utilizing lignocellulosic biomass must be to achieve economic sustainability and reliability in the integrated production of biofuels and co-products [187, 188]. The leading biochemical routes to biofuel production strategies are today based on the fermentation of monosaccharides produced by hydrolysis of the carbohydrate polymers in cell walls of plant biomass [189]. In the context of nanocellulose production, the use of fermentable hydrolysates following nanocellulose production is crucial for enhancing the value proposition of the integrated biorefinery by maximizing transformation of biogenic carbon into desirable products, consistent with modern biorefineries. Thus, the weak and strong acid hydrolysis nanocellulose production schemes that are commonly employed for nanocellulose production are not compatible with this paradigm, considering that the acidic conditions enable loss of sugars in the form of inhibitory degradation products and can entail expensive neutralization steps. Opportunities for production of both nanocellulose and biofuels using methods that are compatible with second-generation biorefinery technology were first reported by Zhu and co-workers at the USDA Forest Products Laboratory [137], who showed that enzymatic hydrolysis could provide CNFs and a soluble sugar stream amenable to downstream fermentation.

At the National Renewable Energy Laboratory (NREL), we also proposed that enzymatic hydrolysis could provide a solution for integrated production of biomass nanocellulose and biofuels. In the classically reported biomass conversion process, hydrolytic and oxidative enzymes with high specificities work to efficiently depolymerize polysaccharides to produce high-quality sugars that are well-suited to downstream fermentation and/or catalytic upgrading [190]. Other advantages embedded in this classical process are ensured by the high degree of specificity displayed by enzymes, compared to chemical catalysts. For example, it is well known that various enzymes target functional groups, or even larger regions of carbohydrate substrates with high selectivity [191].

Relative to chemical production methods, there are relatively few studies of enzymatic nanocellulose production methods in the literature, although this topic has gained new interest of late. Janardhnan and co-workers were perhaps the first to show that treating kraft pulp with a fungal culture prior to mechanical refining improved the yield of microfibrillated cellulose [192]. Soon thereafter, purified endoglucanases were reported to enhance the production of both CNFs and CNCs from softwood pulps [118, 119]. This result was later confirmed and expanded by Filson et al., using recycled softwood pulp as a feedstock. [193]. Xylanase treatments were also applied [135, 136]. Unfortunately, this process strategy resulted in low yields of fermentable sugars, which is not encouraging for biofuel/nanocellulose co-production strategies. Furthermore, energy savings for nanofibrillation by these treatments is significant, but not sufficient for economic CNF production.

Additional studies have focused on refinement of the integration of enzymatic treatment, now using both endoglucanases and exoglucanases following mechanical processing and acid hydrolysis steps. Studies have demonstrated enabled production of nanocellulose of various sizes and aspect ratios [194, 195]. The opportunity for the co-production of nanocellulose and biofuels using enzymatic strategies was first realized by Zhu and co-workers [137]. This work demonstrated the concept of co-production of fermentable sugars and cellulose nanomaterials using commercial cellulase formulations and kraft hardwood pulp as a feedstock. In the years following these foundational studies, additional work has been reported regarding the use of commercial enzyme formulations to achieve production of nanocellulose from a diversity of feedstocks, such as banana peel [196, 197], corrugated packaging [198], soybean straw [199], and kraft pulps [200–202].

**Fig. 9:**
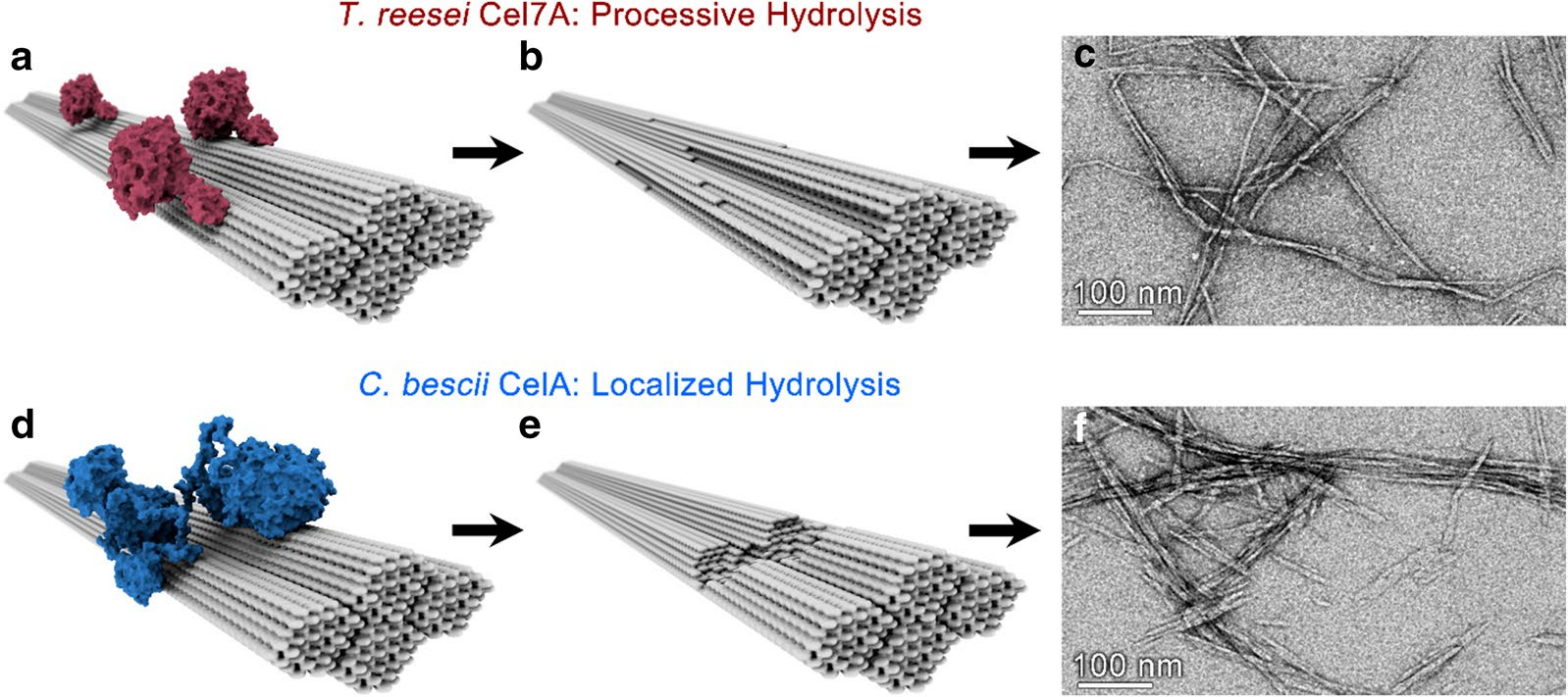
Cellulase enzymes with different degradation mechanisms alter the characteristics of the nanocellulose products. **a–c** T. r*eesei* Cel7A performs processive hydrolysis which results in high aspect ratio digestion products resembling CNFs. **d**–**f** CelA from *C. bescii* tends towards localized hydrolysis which facilitates intra-fibril fragmentation and results in production of more uniform fragments with aspect ratios typical of CNCs. Adapted from Yarbrough et al. [190] with permission. Copyright © 2016, American Chemical Society

The vast diversity of cellulolytic enzyme systems found in nature presents an expansive design space for optimization of cellulase cocktail formulations that are tuned not only for high yield of desired products (sugars), but also for the specific characteristics of the resultant nanocellulose. We recently compared the performance of the ubiquitous free enzyme system of *Trichoderma reesei* to that of hot springs bacterium, *Caldicellulosiruptor bescii,* which contains complexed enzymes equipped with several catalytic domains [203]. The study revealed that bacterial enzyme systems not only outperformed the fungal system in terms of overall conversion, but also produced more uniform nanocellulose particles. This result was attributed to the difference in degradation mechanisms employed by the two systems: the free fungal enzyme system performs a more global, processive hydrolysis; whereas the complexed bacterial system tends to perform localized hydrolysis, which can lead to a “pit-digging” behavior (Fig. 9).

**Fig. 10:**
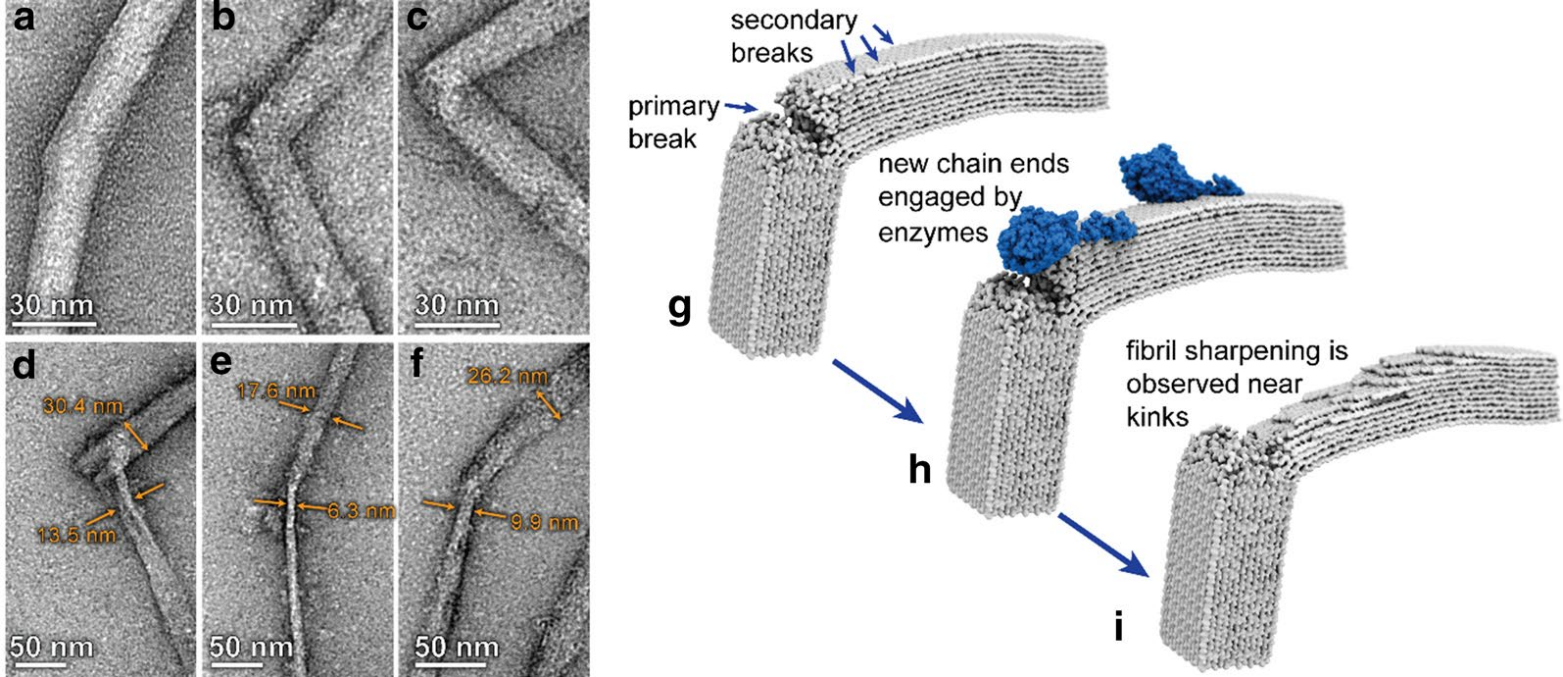
Cel7A preferentially initiates hydrolysis at mechanically induced defects in cellulose nanofibrils. **a**–**c** TEM images of *Cladophora *cellulose nanofibrils exhibiting defects. **d**–**f** TEM images of defect sites in *Cladophora *cellulose nanofibrils following partial digestion by Cel7A. **g**–**i** Schematic depiction of the process by which processive cellobiohydrolases engage molecular defects to produce “sharpened” morphologies near the defect site. Adapted from Ciesielski et al. [204] with permission. Copyright © 2019 National Academy of Sciences

Cellulose is often conceptualized as consisting of regions of a high degree of molecular order separated by localized regions of disorder, as discussed earlier. Recently, it was demonstrated that such disordered regions can arise from the concentration of mechanical stress [204]. The study used nanomechanical manipulation to apply stress to cellulose nanofibrils using contact mode AFM, which resulted in the formation of kink defects in the fibrils. Molecular simulation of the process showed that the defect regions were highly disordered and included breakages in the glucan chains that processive cellulases could use to initiate hydrolysis (Fig. 10). We note that these disordered regions are also more accessible to mineral acids, which is the likely mechanism for preferential acid hydrolysis at these locations. Shortly thereafter, Novy et al., demonstrated a similar effect at larger length scales [205]. This study used fluorescence-tagged carbohydrate-binding modules in tandem with electron microscopy to show that large dislocation regions in whole pulp fibers were preferentially attacked by cellulase enzymes. The microscale dislocations at the scale of whole pulp fibers likely contain a large population of molecular and macromolecular defects, as investigated by Ciesielski et al. [204]. Collectively, these findings suggest the opportunity to co-optimize mechanical deconstruction and enzyme functionalities for desired nanocellulose characteristics and yields, thereby further expanding the design space of enzymatic production strategies.

### LPMOs for CNC and CNF production

Complex mixtures of enzymes (LPMOs, endoglucanases, and xylanases) have been shown to be effective in the production of dispersed CNFs [206–208]. A recent published study on using LPMOs to produce nanocellulose [208] clearly demonstrated that a combination of LPMOs and xylanase enzymes resulted in improved nanofibrillation of kraft pulps. Later, Koskela et al., demonstrated that CBM-free LPMOs are less focused and thus act in a more dispersed manner compared to the CBM-containing enzymes, which leads to greater fiber thinning and better surface charge dispersion [207]. The same study further showed that LPMOs containing fungal CBMs produced fragmented, soluble products, which is likely due to the binding preferences of the fungal CBM 1 (i.e., specific foci on the crystalline regions of cellulose). Valenzuela et al. investigated the impact of substrate crystallinity on the activity of bacterial LPMOs with the objective of identifying substrate and process conditions that were favorable for CNM production [209]. The study showed that the LPMOs used exhibited higher activity on more crystalline substrates and that the enzymatic treatment enhanced nanofibrillation during subsequent mechanical processing.

These studies demonstrate relatively low yield of fermentable sugars due to LPMO-induced oxidation of the C1 carbon of glucose and cellobiose, resulting in the formation of nonfermentable aldonic acids, which is not a favorable outcome in the context of integrated biorefining. If a high yield of fermentable sugars is indeed an intended goal, it seems that LPMO-containing mixtures would benefit from cellobiohydrolases; however, such a formulation has not been reported to our knowledge. Although LPMOs combined with endoglucanase produce dispersed CNFs effectively [207], it is unlikely that these enzyme mixtures can produce CNCs in the absence of other hydrolytic agents. This is because LPMOs act primarily on the exposed surface regions of cellulose, where they leave residual surface carboxylation resulting in effective fiber dispersion. Furthermore, the surface modification activity of LPMOs does suggest their utility for tuning the chemical functionality of nanocellulose products, which may prove to be a central production strategy for targeted end use applications.

## Drying and dewatering of cellulosic nanomaterials

### Drying techniques for CNMs

Generally, CNMs are in aqueous state when they are produced, which loosens and breaks the interfibrillar hydrogen bonding. As fibrillation proceeds, cellulose surface area is increased and more and more hydroxyl groups are exposed to water molecules, which leads to a high water-retention capacity. This is clearly seen from the increase in water-retention value of CNFs with fibrillation [210]. Water usually accounts for more than 95% of total mass of CNM suspension. A large amount of water molecules is restrained in the interfibril meniscus (freezing water and freezing bound water) as well as in the thin layer on fibril external surfaces (non-freezing water) [211, 212]. The high water content in CNM suspension makes material handling difficult and negatively impacts applications. Moreover, it also significantly increases cost for shipping CNM suspensions that contain primarily water. To address this issue, various dewatering and drying techniques have been established and studied to dehydrate CNMs.

### Air/oven-drying

Air/oven-drying (AD/OD) is a solvent evaporation process. It is generally used to prepare CNM films following a pressure filtration or solution casting procedure [213–215]. In some cases, solvent exchange is introduced before drying. As water evaporates, a capillary pressure gradient built by intensive surface tension pulls cellulosic fibrils close enough to form hydrogen bonding to result in a sheet-like structure that is densely packed with randomly oriented interwoven fibrils [216]. A solvent-exchange process before AD/OD may lessen aggregation caused by water evaporation and lead to a more porous structure of air/oven-dried CNMs, thus the products can be more permeable to both gas and water molecules. Toivonen et al. fabricated a CNF aerogel membrane by filtration, solvent exchange with 2-propanol and octane, and subsequent ambient drying [217]. Unlike a highly aggregated and condensed CNF film produced by drying directly, the CNF aerogel membrane possessed mesoporosity, high specific surface area, and low density.

Direct AD/OD is the simplest method for desiccating CNM, but poor redispersibility as a result of strong interfibrillar aggregation or coalescence caused by AD or OD, which substantially affects CNM performance for many commercial applications [218]. On the other hand, AD/OD is an optimal choice among other drying techniques to retain the chiral nematic liquid crystalline order inherent of CNCs in solid form [219]. A dilute isotropic suspension of CNCs is a transparent fluid and normally stabilized by anionic surface groups. As water evaporation proceeds, a critical concentration is reached to form an anisotropic, ordered chiral nematic phase. As the concentration of CNCs continues to increase, the anisotropic phase gradually takes over from the isotropic phase and ends in trapping the chiral nematic organization in an iridescent film [220]. Further information about self-assembling behavior of CNCs during the AD/OD has been summarized and discussed elsewhere [221].

### Freeze-drying

Freeze-drying (FD) has been widely used to remove water from CNM suspension with minimum impact on CNM morphology; as well as to tune the hierarchical porous structure of CNM aerogel or foam products [222]. It typically includes three stages: (1) freezing stage, (2) primary drying stage, and (3) secondary drying stage [223]. In the freezing stage, water is phase transferred into ice crystals, where CNM and water molecules are segregated. Then ice crystals are sublimed during the primary drying stage. In the third stage, non-freezing water is removed by heating the product under vacuum, where agglomeration may occur [223]. Freeze-drying has its inherent complex effects on product morphology, which has to do with freezing rate and solvent media; as well as characteristics of the CNM, such as dimension, surface charge, and especially suspension concentration. Under the same freezing condition, the morphology of a freeze-dried CNF is highly concentration dependent. Ultrafine nanofibers in submicron scale as well as ribbons and flake/film-like structures can be obtained by adjusting initial concentration of the suspension. Han et al. investigated the effect of suspension concentration, particle size, and surface charge on the morphology of CNFs and CNCs prepared by freeze-drying (at – 75 °C) [224]. In a dilute suspension (0.05 wt% or lower), both CNCs with widths of several nanometers and CNFs with widths of tens of nanometers were assembled into ultrafine fibers with submicrometer widths (500 to 1000 nm). Under this condition, samples with higher surface charges are likely to have smaller diameter due to stronger mutual repulsion when self-assembled into submicron fibers. Increasing the suspension concentration to 0.1 wt% resulted in ribbon and sheet-like structures coexisting in the dried foam of CNCs and CNFs, indicating a transition from ultrafine fibers to membrane structure. Further increasing suspension concentration to 0.5 to 1 wt% resulted in lamellar structures composed of aligned thin membranes in both dried CNC and CNF foams. However, a rougher surface with visible dendrites was observed in CNF foams, compared with a homogenous and smooth surface observed in CNC foams. This concentration- and surface charge-dependent morphology of freeze-dried CNMs has also been shown by Jiang et al. [225]. According to their research, there is a critical fiber-to-film transformation concentration for both CNCs and CNFs; however, it is one order of magnitude lower for thinner but longer Tempo-mediated oxidation CNFs (T-CNFs) than rod-like S-CNCs. They attributed this lower critical transition concentration of T-CNFs to the presence of the greater amount of carboxyl groups (1.29 mmol/g) on T-CNF surface by TEMPO-mediated oxidation than the amount of half-ester sulfate groups (0.24 mmol/g) on S-CNC. The much stronger interfibril hydrogen bonding capability of T-CNFs makes it ready to assemble to a greater extent than S-CNCs.

*Tert-*Butanol has been proved to have an inhibitory effect on CNM self-assembling in both lateral and longitudinal directions. Finer fibrils can be obtained from a suspension containing freeze-dried *tert*-butanol than those from an aqueous suspension. It is hypothesized that the lower degree of self-assembling of *tert*-butanol freeze-dried CNFs can be attributed to the steric hindrance of *tert*-butanol-bound CNF surface; as well as the different hydrogen bonding capacity between *tert*-butanol and water with nanocellulose [225]. Information on morphology and characteristics of FD samples in *tert*-butanol suspension or partially exchanged with butanol have been summarized [226].

### Supercritical CO_2_-drying

Supercritical CO_2_-drying (SCD) was commonly used to fabricate nanoporous CNF aerogels, to avoid collapse of the porous structure driven by the surface tension of solvent. At supercritical conditions, surface tension is zero, which prevents the pore structure from collapsing [227]. SCD typically consists of four steps: (1) replacing the solvent in CNF suspension with an intermediate liquid that is both water and liquid CO_2_-miscible; (2) replacing the intermediate liquid with liquid CO_2_; (3) pressurizing and heating liquid CO_2_ to the supercritical conditions, and (4) eliminating supercritical CO_2_ by decompression [223]. Acetone, methanol, and ethanol are common intermediate liquids to completely exchange water. Liquid CO_2_ is chosen as the drying medium because of its conditional advantage over other media, such as low critical temperature (31.0 C°) and moderate critical pressure (7.37 MPa), which is favorable for CNFs because the degradation of cellulose can be avoided [228]. Nanometric fibrils can be well preserved after SCD; however, the resultant CNFs still have larger diameters than their original ones in never-dried suspension [223]. Products obtained by SCD have greater specific surface areas [214, 229–231]. However, it is worth noting that few studies were reported using SCD to desiccate CNC suspensions. This may be attributed to the failure of completely replacing water with an intermediate liquid such as acetone and ethanol when attempting to dry a CNC suspension with SCD [223].

Both freeze-dried and SCD are widely used to tailor the hierarchical porous structure of CNMs, which has been amply reviewed [232].

### Spray-drying

Spray-drying (SD) is a method of converting CNM suspension or slurry into powder by rapid water evaporation using a hot gas. During the spray-drying process, the pre-concentrated CNM suspension is first atomized into droplets, followed by dehydration in a stream of hot gas through a dryer chamber. The size of spray-dried CNCs and CNFs are in the range of several microns. For long CNFs, irregular rod-like particles with small fibrils adhering to the particle surface were obtained after spray-drying. For CNCs with much smaller sizes and a narrower size distribution, spherical particles were obtained [223]. The dehydration process of spray-drying in a dryer chamber is similar to that of oven-drying, and strong aggregation occurs during spray-drying. However, the powder forms of spray-dried CNMs are more favorable for applications as tablet excipient and as reinforcing polymers [233–235].

### Redispersibility of dried CNMs

The key concerns about drying/dewatering of CNMs focus on how to restore their dimensions and properties at the nanoscale. As discussed above, CNMs dried by diverse techniques all undergo irreversible aggregation at different levels. It is unlikely to get the dried CNMs thoroughly redispersed into their original state with a mild mechanical disintegration without any additives. However, SCD and FD are much more reliable for preserving initial dimensions than SD and AD/OD. As a result, CNMs obtained from SCD and FD redispersed much more quickly than films from AD or powder from SD [236]. For AD/OD films, the interlaced fibrils have been firmly held together by strong hydrogen bonding. Water penetrates more slowly into a thicker film than into a porous foam obtained from FD or SD. As for micrometric granules from SD, when immersed in water, a gel layer will form at the granule surface surrounding a relatively large core of dry CNM. This leads to a slow water penetration and thus a poor redispersibility [219]. The mechanical energy input for redispersion of dried CNMs can be extremely high.

Improvement of redispersibility of CNMs can be achieved by surface modification or by additives, which can interfere with the formation of interfibril hydrogen bonding. It has been demonstrated that the addition of salt, such as NaCl, can improve redispersibility for both acid form S-CNCs (H^+^-CNC) and CNFs in water [219, 237], but with completely different mechanisms. In the case of H^+^-CNCs from concentrated sulfuric acid hydrolysis, a large amount of SO_3_^−^ on the surface tends to adsorb Na^+^ thus reducing the probability of forming hydrogen bonding. As for CNFs, ion–dipole interactions between hydroxyl groups of cellulose and the salt play a key role in preventing interactions between interfibril hydroxyl groups. Other water-soluble additives, such as carboxymethyl cellulose, fish-derived gelatin, and maltodextrins, recently have also been shown to have a positive effect on enhancing the redispersibility of dehydrated CNFs [238–240].

### Dewatering of CNMs

Partial dewatering is an alternative for complete drying to avoid drying-caused aggregation or hornification, and at the same time lowers the cost for transportation. Solvent evaporation and conventional mechanical dewatering methods, such as vacuum/pressure filtration and centrifugation, are common methods used in the laboratory. However, these processes become very time consuming and energy intensive when dealing with CNMs with large specific surface area and high water-retention capacity. In practice, they are suitable for preconcentrating CNM suspension and are often combined with other methods, such as mechanical pressing and absorbing, for further dewatering [241, 242]. Some additives and auxiliary methods have been proven to increase dewatering efficiency.

### Electro-assisted filtration

Electro-assisted filtration, also known as electrofiltration, recently has been demonstrated to be effective in improving dewatering rate of CNCs. In electrofiltration, an electric field is applied to influence the filtration of negatively charged CNCs by inducing an electrophoretic force in the direction of the anode, thereby influencing the filter cake growth. At the same time, it also induces motion of the fluid in the direction of the cathode, which drives solid–liquid separation [243, 244]. In this process, the electric field is a dominant factor influencing the dewatering rate, exceeding the effect of modifying suspension conditions. The filtrate flow rate increases significantly when an electric field is applied. Furthermore, it increases with increasing strength of the electric field. Because the filter cake can be counteracted by the electric field, thereby decreasing the filtration resistance. In addition to the electric field, increasing temperature by heating is also a positive factor for improving filtrate flow rate [245].

### Other dewatering methods

Other dewatering methods, such as adding counter ions, polymers, and adjusting pH, have been studied to improve dewatering efficiency of CNMs. Flocculation caused by adding salt, thereby causing a charge neutralization, can positively affect drainage of a CNF suspension [246]. When a salt, such as NaCl or CaCl_2_ was added to a CNF suspension, aggregation and sedimentation of nanofibrils occurred as a result of weakening electrostatic repulsion, which was caused by compression of the electric double layer. Significant increases in the dewatering rate of CNF suspensions were observed when the ζ-potential was changed from negative to near neutral by adding salt. Bivalent cations with higher ionic strength were more effective for improving the drainage of CNF suspensions than monovalent cations.

The dewatering behavior of CNFs mixed with wood particles of different sizes or specific surface areas was investigated [247]. Results showed that dewatering rate by pressure filtration of CNFs mixed with wood particles was significantly improved using particles with high specific surface area compared with pure CNFs alone. However, the amount of water removal of CNFs mixed with wood particles is lower than pure CNFs. The study hypothesized that the absorbed water in CNFs turned into free water as a result of physical contact between CNFs and wood particles, which increased the rate of dewatering.

Unbound water can be released from a colloidal CNF-based gel by two-component solid demixing and differential autoflocculation of nanofibrils under conditions of ultralow shear [248]. It was demonstrated that by applying a high shear for a short period of time before applying an ultralow shear, a large amount of free-flowing water can be released from the multicomponent CNF suspension. It is worth noting that flocculation of CNFs induced by adding unstable colloidal particles is not strong enough to cause dewatering. Applying ultralow shear is also necessary in this procedure. This is a low-energy dewatering process; however, the removal of unstable colloidal particles remains a problem in practical use. In subsequent work, acid dissociation of surface-bound water on CNFs was also revealed by adsorption of calcium carbonate nanoparticles under the application of ultralow shear [249].

Drying or dewatering of CNFs/CNCs will lead to a significant saving in transportation cost, but massive energy consumed during the desiccation procedure is inevitable. Furthermore, the CNFs/CNCs usually cannot recover their original properties using traditional drying method. Partial dewatering seems to be a trade-off between energy consumption and transportation cost. Several other dewatering methods involve additives to maintain redispersibility in water. However, efficient removal of these additives becomes a secondary problem. Facile and scalable techniques for dewatering and drying of CNFs/CNFs remain as a technical challenge.

## Conclusions and future outlook

Economic and sustainable production of plant-based NPMs, especially CNMs, remains one of the main barriers to commercialization of NPM-based products. Exploration of the utility of lignin-based NPMs, such as LNPs, is still at an early stage, in terms of both production and utilization. With continued research, we expect that sustainable and commercially viable LNP production techniques will be developed. Especially interesting are techniques that can be integrated into the plant biomass fractionation process, such as AHF [43, 45, 66], or techniques that could use spent liquors from commercial wood pulping directly without purification and drying in between. Surface functionality of LNPs can further facilitate the development of novel LNP-based products in different new markets for lignin valorization. Examples of these new products are particles for drug delivery [62, 87], enzyme immobilization [83], or sun screen applications [56, 250], and their dispersing ability and UV-protective properties make LNPs attractive for paints, coatings, and cosmetics. In many applications, production of LNPs with visually appealing color, unlike the dark brown color of commercial technical lignin, are favorable. Various applications have very different demands for the LNP material, hence future research should also focus on comparing methods and finding the best particle preparation method for different applications.

Significant research effort in both production and utilization of cellulose-based NPMs (i.e., CNMs) has been deployed. Production processes using concentrated mineral acid hydrolysis, pure mechanical fibrillation, endoglucanases, and TEMPO-mediated oxidation treatments have been scaled-up for application research. Addressing concerns over environmental impact and production economics due to high energy input and difficulties in chemical recovery associated with these early CNM production processes will be a great challenge. Recent research trends and funding, however, are heavily focused on new CNM-based product development using CNMs from these early production techniques. Although new product development is important, addressing CNM production economics and sustainability is a critical prerequisite for commercialization. Some new research focus is needed toward the novel CNM production processes that can address low-cost and sustainable production. In this progress report, we have provided a few promising process perspectives, such as concentrated dicarboxylic acid hydrolysis for integrated production of CNCs with CNFs, and the use of CNWs to substitute for most CNC applications.

Moreover, enzymatic treatment remains highly attractive for CNM production. Post-fibrillation endoglucanase treatment can be effective to break up fibril aggregates for producing CNWs. With the development of novel enzymes, such as new bacterial cellulases and LPMOs, new concepts for utilizing specialty enzymes, such as integrating CNM production into a biorefinery concept as presented in this report, are worthy of consideration.

Dewatering is another challenge for transportation, drying, and applications of CNMs in hydrophobic media. Novel drying approaches that can avoid CNM aggregation and improve CNM redispersibility and drying energy efficiency also need to be developed. For large-volume applications, such as for papermaking, on-site production of CNFs or CNMs is recommended for potential savings in production and transportation. Redispersion of LNPs after drying is not a problem, and dewatering is also less of a problem than for CNMs.

Finally, future product development should focus on [251] (1) low-cost products with marginal performance improvement to substitute for existing petroleum-based products (i.e., drop-in market product); (2) novel usages with unique performance properties engineered to display orders of magnitude performance improvement, and (3) low-cost and large-volume applications from the forest management perspective.

## Data Availability

None.

## Abbreviations

AHF: Acid hydrotropic fractionation
BADGE: Bisphenol A diglycidyl ether
BEP: Bleached kraft eucalyptus pulp
CDF: Combined delignification factor, Eq. (3)
CHF_X_: Combined hydrolysis factor for xylan (X), Eq. (5)
CHF_G_: Combined hydrolysis factor for cellulose (G, glucan), Eq. (1)
CNCs: Cellulose nanocrystals, refers to a class of individually separated short crystalline cellulosic nanoparticles with aspect ratios of approximately 30 or smaller and good crystallinity. CNCs are often produced by concentrated acid hydrolysis unless high cellulose crystallinity is demonstrated
LCNCs: CNCs containing lignin
S-CNCs, DC-CNCs, O-CNCs, M-CNCs: CNCs produced by hydrolysis using concentrated sulfuric (S), dicarboxylic (DC), oxalic (O), maleic (M) acid, respectively
CNFs: Cellulose nanofibrils that refer to nanoscale cellulosic fibrils that can be individually separated or physically entangled or interconnected as fibril networks
LCNFs: CNFs containing lignin
DC-CNFs, M-CNFs: CNFs produced by dicarboxylic (DC) acid, maleic (M) acid hydrolysis
T-CNFs: TEMPO-mediated oxidation (T)
CNMs: Cellulose-based nanomaterials
LCNMs: CNMs containing lignin
CNWs: Cellulose nano-whiskers, refer to a class of individually separated short cellulosic nanoparticles with a morphology similar to CNCs and aspect ratios of approximately 30 or smaller. Crystalline CNCs is a subcategory of CNWs
CSR: Cellulosic solid residues from (acid or enzymatic) hydrolysis of cellulosic materials
DCA; OA; MA: Dicarboxylic acid (DCA); oxalic acid (OA); maleic acid (MA)
DP: Cellulose degree of polymerization
L_R_: Fraction of lignin retained on fractionated lignocellulosic solids, Eq. (4)
X_R_: Fraction of xylan retained on fractionated lignocellulosic solids, Eq. (6)
LNPs: Lignin nanoparticles
NPMs: Nanoscale (lignocellulosic) polymeric materials
KL; AL; OSL: Kraft (K), alkali (A), and organosolv (OS) lignin
AD; FD; OD: Air-drying; freeze-drying; oven-drying
SCD; SD: Supercritical-drying; spray-drying
SET-LRP: Single electron transfer-living radical polymerization
